# Environmental variability and heavy metal concentrations from five lagoons in the Ionian Sea (Amvrakikos Gulf, W Greece)

**DOI:** 10.3897/BDJ.4.e8233

**Published:** 2016-11-01

**Authors:** Katerina Vasileiadou, Christina Pavloudi, Ioanna Kalantzi, Eugenia T. Apostolaki, Giorgos Chatzigeorgiou, Eva Chatzinikolaou, Evangelos Pafilis, Nafsika Papageorgiou, Lucia Fanini, Spyridon Konstas, Nina Fragopoulou, Christos Arvanitidis

**Affiliations:** ‡ Institute of Marine Biology, Biotechnology and Aquaculture, Hellenic Centre for Marine Research, Heraklion, Crete, Greece; § Department of Biology, University of Patras, Patras, Greece; | Department of Biology, University of Ghent, Ghent, Belgium, Department of Microbial Ecophysiology, University of Bremen, Bremen, Germany; ¶ Institute of Oceanography, Hellenic Centre for Marine Research, Heraklion, Crete, Greece; # Department of Biology, University of Crete, Heraklion, Crete, Greece; ¤ Amvrakikos Management Body, Arta, Greece

**Keywords:** environmental variables, heavy metals, Amvrakikos Gulf, lagoons

## Abstract

**Background:**

Coastal lagoons are ecosystems of major importance as they host a number of species tolerant to disturbances and they are highly productive. Therefore, these ecosystems should be protected to ensure stability and resilience. The lagoons of Amvrakikos Gulf form one of the most important lagoonal complexes in Greece. The optimal ecological status of these lagoons is crucial for the well-being of the biodiversity and the economic prosperity of the local communities. Thus, monitoring of the area is necessary to detect possible sources of disturbance and restore stability.

**New information:**

The environmental variables and heavy metals concentrations, from five lagoons of Amvrakikos Gulf were measured from seasonal samplings and compared to the findings of previous studies in the area, in order to check for possible sources of disturbance. The analysis, showed that i) the values of the abiotic parameters vary with time (season), space (lagoon) and with space over time; ii) the variability of the environmental factors and enrichment in certain elements is naturally induced and no source of contamination is detected in the lagoons.

## Introduction

Coastal lagoons are ecosystems of great economic and ecological importance. They are highly productive systems (Knoppers 1994) thus, intensively exploited (e.g. aquaculture, fisheries) (Lirman et al. 2007, De Pascalis et al. 2012). Lagoons are offering protection and food to several species (Barnes 1980). Nevertheless, they are explicitly fragile as they are naturally enriched, temporally and spatially unstable and vulnerable to human and natural pressures (Cañedo-Argüelles et al. 2012).

Lagoons are coastal aquatic systems separated from the sea by sediment barriers and connected to it through channels (Barnes 1995). They are characterized by strong salinity gradients which are dependent on the seawater and freshwater inflows. Hydrodynamics are responsible for the temporal and spatial shifts of the environmental factors and the formation of many different chemical gradients (Loureiro et al. 2006, Nicolaidou et al. 2006). The organically enriched sediments often trigger dystrophic crises events (Guelorget and Perthuisot 1983, Sfriso et al. 1992, Nicolaidou et al. 2006), inducing mass mortality events of populations (Vignes et al. 2009). Lagoonal waters may receive significant quantities of nutrients, heavy metals and pollutants (Sfriso et al. 1992, Cobelo-García et al. 2012, Prado et al. 2014). Their shallowness along with the synergistic effect of the wind are responsible for the resuspension of the nutrients from the sediments and their further distribution to the water column through mixing (Diamantopoulou et al. 2008, Pérez-Ruzafa et al. 2011).

The human activities occurring inside and around the lagoons are an additional source of pressure to these systems. The hydrological balance of the lagoons can be altered according to the needs dictated by the uses of the area (Duck and da Silva 2012). Thus, river inflows can be favored or blocked over the sea inflows causing changes on the hydrological/physico-chemical regime and, subsequently, on the environmental and biological processes. Moreover, contaminants are introduced into the systems through wastewater, human activities and freshwater runoffs from the land (Specchiulli et al. 2008, Pérez-Ruzafa et al. 2011, Guerra et al. 2013). Inflows ofpollutants can change the physico-chemical profile of the lagoons. Non-natural enhancement of nutrient concentrations has been connected to hypoxic or even anoxic events, as well as eutrophication events (Loureiro et al. 2006, Gianni and Zacharias 2012, Cañedo-Argüelles et al. 2012, Guerra et al. 2013). Eutrophication induces serious shifts to the nutrient cycles, which may destabilize the ecosystem (De Casabianca et al. 1997, Orfanidis et al. 2005, Vignes et al. 2009).

Transitional water systems are also threatened by the introduction of heavy metals. Heavy metals are accumulated on the surface sediments of the lagoons (Sfriso et al. 1992), thus their concentration is affected not only by the anthropogenic activities and the water inflows but also by the grain size distribution (Bellucci et al. 2002). Mobilization of the elements is achieved through sediment resuspension caused by both natural and non-natural processes or through decomposition of organic material (Donazzolo et al. 1984, Sfriso et al. 1992) and may risk public health. Therefore, assessment of heavy metal concentrations in the lagoonal sediment is of major importance (Acevedo-Figueroa et al. 2006).

The lagoonal complex of the Amvrakikos Gulf is located in western Greece. They were formed by the flowing activity of the rivers Louros and Arachthos and they are considered as one of the most important and productive lagoonal complexes in Greece. Gilthead sea bass, mullets, gobies and eels are yielded in these lagoons through extensive aquaculture (Katselis et al. 2013). The lagoons are included in the Natura 2000 network, they are protected under the Ramsar Convention and no source of intensive anthropogenic disturbance has been reported (Reizopoulou et al. 1996, Kormas et al. 2001, Reizopoulou and Nicolaidou 2004). Seasonal changes of freshwater inflows are responsible for the temporal shifts in nutrient concentrations as well as in the other abiotic parameters (Kormas et al. 2001). The lagoons are shallow, hence the water column is well mixed during most of the year (Reizopoulou and Nicolaidou 2004).

The present study attempts to test whether the environmental parameters as measured in five lagoons from the Amvrakikos Gulf lagoonal complex, vary with time (season), space (lagoon) and lagoonal gradient (location) and to compare the results with those of previous studies in the area.

## Project description

### Personnel

Katerina Vasileiadou, HCMR/University of Patras (sample collection, sample analyses, data management), Christina Pavloudi, HCMR/University of Crete (sample collection, sample analyses, data management), Ioanna Kalantzi, University of Crete (sample analyses), Georgios Chatzigeorgiou, HCMR (sample collection), Eva Chatzinikolaou, HCMR (sample collection), Nafsika Papageorgiou, University of Crete (sample collection), Eugenia Apostolaki, HCMR (sample collection), Evangelos Pafilis, HCMR (sample collection), Lucia Fanini, HCMR (sample collection), Spyros Konstas, Amvrakikos Wetlands Management Body (sample collection), Nina Fragopoulou (project coordinator, sample collection), Christos Arvanitidis, HCMR (project coordinator, sample collection)

### Funding

This work was supported by the LifeWatchGreece infrastructure (MIS 384676), funded by the Greek Government under the General Secretariat of Research and Technology (GSRT), ESFRI Projects, National Strategic Reference Framework (NSRF).

## Sampling methods

### Sampling description

**Figure 1.**
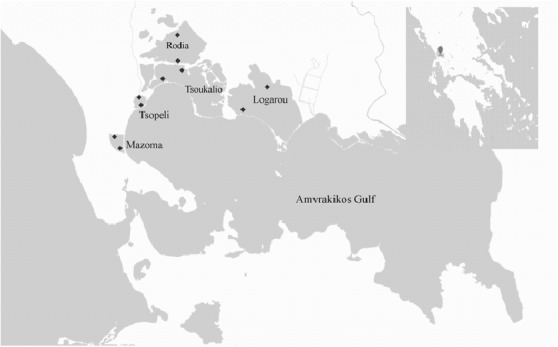
Map of the stations of the lagoonal complex of Amvrakikos Gulf.

The sampling area is located in Amvrakikos Gulf (W Greece) (Fig. 1). Five lagoons were studied: Logarou (39° 02΄N; 20° 54΄Ε), Tsoukalio (39° 03΄N; 20° 48΄Ε), Rodia (39° 4΄N; 20° 48΄Ε), Mazoma (39° 00΄N; 20° 44΄Ε) and Tsopeli (39° 02΄N; 20° 46´E). Logarou lagoon is extended over a surface of 28,000 ha and is the largest one out of the five lagoons sampled. However, it is rather shallow as the water depth does not exceed 1.5 m. Tsoukalio on the southern part is separated from the sea by sand barriers with narrow openings allowing limited water exchange, while on the northern part it is connected to Rodia lagoon through a narrow opening of 15 m. Rodia lagoon is an internal lagoon and lacks any connection with the sea. Mazoma is a small lagoon formed in the opening of Louros river with a surface area of 300 ha. Tsopeli is the smallest of the five lagoons (approx. 120 ha surface) without any obvious source of pollution.

In each lagoon two sampling stations were chosen: one located near the opening connecting the lagoon to the sea, and another one in its inner part (Fig. 1). Four samplings were carried out: September 2010, February 2011, May 2011 and July 2011.

Salinity, temperature, oxygen concentration and pH were measured in the water column, while temperature, Redox potential and conductivity were measured in the first two centimeters of the sediment on site. All measurements were taken by means of a portable multi-parameter (WTW Multi 3420 SET G). Three replicate samples of one litre volume from the water column and three of sediments were randomly taken from each station. The water samples were used to estimate the nutrient concentrations in the water column: phosphate (PO_4_), nitrate (NO_3_), nitrite (NO_2_), ammonium (NH_4_), silicon dioxide (SiO_2_). Moreover, the percentage of labile organic matter (labile OM) concentration was estimated from the sediment samples, the concentrations of chlorophyll-a and particulate organic carbon (POC) in both the water column and the sediments. Additional sediment samples were collected from each station for granulometry. A list of all the parameters measured during the study is presented on Table 1.

The concentration of the Total Reduced Inorganic Sulfur (TRIS) in the sediments was estimated. A volume of eight cubic centimeters was collected from the sediment surface and incubated immediately with 10 ml of zinc acetate solution 20% (w/v). The samples were mixed to ensure the immersion of the total volume of the sediment in the solution and stored at -20 °C.

In addition, sediment samples were processed to determine the heavy metal concentrations from the sampling campaigns of February, May and July of 2011. The samples from the expedition of September 2010 were contaminated and thus not used in the analysis. Therefore, the heavy metal concentrations are not analyzed seasonally and the concentrations were averaged per station.

## Geographic coverage

### Description

Five lagoons were studied (Logarou, Tsoukalio, Tsopeli, Rodia, Mazoma).

### Coordinates

38.64 and 39.16 Latitude; 20.33 and 21.36 Longitude.

## Usage rights

### Use license

Creative Commons CCZero

## Data resources

### Data package title

Benthic communities in Amvrakikos Wetlands: Mazoma, Tsopeli, Tsoukalio, Rodia and Logarou lagoons (September 2010 – July 2011)

### Resource link

http://ipt.medobis.eu/resource?r=zoobenthos_in_amvrakikos_wetlands

### Number of data sets

1

### Data set 1.

#### Data set name

Benthic communities in Amvrakikos Wetlands: Mazoma, Tsopeli, Tsoukalio, Rodia and Logarou lagoons (September 2010 – July 2011)

#### Data format

Darwin Core Archive

#### Number of columns

7

#### Character set

UTF-8

#### Download URL

http://ipt.medobis.eu/resource?r=zoobenthos_in_amvrakikos_wetlands

#### Description

The dataset is available via the MedOBIS (Mediterranean node of Ocean Biogeographic Information System) Internet Publishing Toolkit (IPT) of the Hellenic Centre for Marine Research (HCMR). The data will also be harvested by and made available through the European node of the Ocean Biogeographic Information System (EurOBIS), as well as through the International OBIS database. The dataset is available as a DarwinCoreArchive, all fields are mapped to DarwinCore terms. This publication refers to the most recent version of the dataset available through the IPT server or MedOBIS. Future changes to the dataset due to quality control activities might change its content or structure.

The current publication refers to the MeasurementOrFact source file that is associated with the particular data set.

**Data set 1.** 

Column label	Column description
id	An identifier for the set of information associated with each event.
measurementID	A unique identifier for the record within the data set, auto-incrementing number automatically added by the system.
measurementType	The measured environmental variable.
measurementValue	The value of the measurement.
measurementUnit	The units associated with the measurementValue.
measurementMethod	The method or protocol used (with reference to publication) to determine the measurement.
measurementRemarks	Comments accompanying the MeasurementOrFact.

## Additional information

### Samples processing

The nutrient concentrations in the water column were processed following the protocols by Grasshoff et al. (1983) and Parsons et al. (1984) and measured by means of UV/VIS Spectrophotometer (U-1800, Hitachi). The percentage of labile OM concentration was estimated using the protocols by Loh et al. (2008). The concentrations of chlorophyll-a and POC were assessed in both the water column and the sediments following the methods of Hedges and Stern (1984) and Yentsch and Menzel (1963)measured by means of Fluorometer (TD-700, Turner Design). The granulometry samples were analyzed following the protocols by Gray and Elliott (2009). The samples of TRIS were analyzed following the hot distillation method (Kallmeyer et al. 2004). The final measurement of the TRIS values was based on the method reported by Cline (1969).

The heavy metal samples were treated following the methods described by Kalantzi et al. (2013). Data quality assurance was performed by using one blank and one certified reference material (marine sediment, NCS DC75305 and NCS DC75301) from the China National Analysis Centre in every 6 samples digested. For the samples from the sampling cruise of February 2011, the average recoveries of all elements of NCS DC75305 was 92.61±1.43% (n=33) and of NCS DC75301 was 85.67±4.64% (n=31). For precision assessment, three different sediment samples were analyzed 3 times each and RSD was lower than 12% for all elements except for Cd, which had RSD ~13.5%. The element concentrations in the digestion blanks were typically very low and were subtracted from the sample values. The limits of detection (LOD) of the procedure were calculated by multiplying the standard deviation of the blanks (n=6) by three and were: 0.11 (Li), 0.02 (Be), 15.49 (Na), 12.25 (Mg), 0 (Al), 17.94 (P), 31.46 (K), 16.43 (Ca), 0.04 (Sc), 0.02 (V), 0.3 (Cr), 0.16 (Mn), 24.49 (Fe), 0.03 (Co), 0.35 (Ni), 0.5 (Cu), 0.76 (Zn), 0 (Ga), 0.02 (Ge), 0.14 (As), 0.01 (Rb), 0.06 (Sr), 0.01 (Y), 0 (Pd), 0.02 (Ag), 0.01 (Cd), 0 (Cs), 0.01 (La), 0.01 (Ce), 0 (Pr), 0 (Nd), 0 (Eu), 0 (Sm), 0 (Gb), 0 (Tb), 0 (Dy), 0 (Ho), 0.01 (Er), 0 (Tm), 0 (Yb), 0 (Lu), 0 (Tl), 0.03 (Pb), 0 (Th) and 0 (U) mg/kg.

The rest of the sediment samples were processed in a different analytical run. Average recoveries of all elements of NCS DC75305 was 96.7±2% (n=41) and of NCS DC75301 was 91.6±4.7% (n=39). For precision assessment, three different sediment samples were analyzed 3 times each and RSD was lower than 12% for all elements. The element concentrations in the digestion blanks were typically very low and were subtracted from the sample values. The limits of detection (LOD) of the procedure were calculated by multiplying the standard deviation of the blanks (n=14) by three and were: 4.67 (Li), 0.01 (Be), 35.28 (Na), 15.37 (Mg), 10.76 (Al), 22.78 (P), 68.73 (K), 132.70 (Ca), 0.04 (Sc), 0.01 (V), 0.3 (Cr), 0.46 (Mn), 34.09 (Fe), 0.01 (Co), 2.28 (Ni), 0.38 (Cu), 3.82 (Zn), 0 (Ga), 0.02 (Ge), 0.06 (As), 0.06 (Rb), 0.36 (Sr), 0.01 (Y), 0.01 (Pd), 0.01 (Ag), 0 (Cd), 0 (Cs), 0.03 (La), 0.05 (Ce), 0.01 (Pr), 0.02 (Nd), 0 (Eu), 0.01 (Sm), 0 (Gb), 0 (Tb), 0 (Dy), 0 (Ho), 0 (Er), 0 (Tm), 0 (Yb), 0 (Lu), 0 (Tl), 0.09 (Pb), 0.01 (Th) and 0 (U) mg/kg.

Both the analysis of TRIS and element concentrations were realized in the in the Marine Ecology Laboratory of the University of Crete.

### Statistical analysis

A series of methods were used for the statistical analysis of the data. Matrices of the abiotic parameters over stations and seasons were produced to compare the similarities: i) between all stations over all seasons, ii) between the stations for each season, iii) between the stations over seasons per lagoon, iv) between the stations near to the sea and the stations in the innermost parts of the lagoons. The similarities were calculated using the Euclidean distance and they were used to create nMDS plots. Permutational multivariate analysis of variance (PERMANOVA) (Anderson 2001) was performed to test for the significance of the factors affecting the resulting similarity patterns. A reduced model was used for the permutation of residuals combined with a Type III (partial) sum of squares.

The Principal Component Analysis (PCA) was used to detect the parameters responsible for the patterns observed between the stations. The similarities between the stations were estimated again using the Euclidean distances. For the previously described routines the PRIMER v. 6.1.8 (Clarke and Gorley 2006) software was used. Significance of the PCA analysis was tested with the Kaiser-Guttman approach (Jackson 1993). The approach calculates the ratio λ between each eigenvalue and the average of all eigenvalues. The information from the components with λ>1 is the one retained. The value to consider a variable as strongly associated with any of the eigenvectors was set at higher than 0.5 or lower than -0.5.

Moreover, linear regression was applied between the values of salinity (independent variable) and those of the abiotic parameters (dependent variables) (Xu et al. 2008), to identify the most important parameters per lagoon affected by the seasonal inflows or evaporation. The Kolmogorov-Smirnov normality test was applied on the abiotic parameters and for those non-fitting the normal distribution, the logarithmic transformation was used prior to enter analysis.

The heavy metal concentrations in the sediments were measured per station and the outcomes were compared against the Threshold Effect Level (TEL) using the standards implemented by the Canadian Freshwater Sediment Guidelines (Burton 2002) and against the Sediment Quality Guidelines (SQG) implemented by the United States Environmental Protection Agency (USEPA) (Pekey et al. 2004). Moreover, the geo-accumulation index (I_geo_) (Muller 1969) was implemented in order to estimate the heavy metal concentrations above background concentrations. The index values are ascribed to seven enrichment classes. Each class is attributed to an estimate of the heavy metal pollution level (Muller 1969). For the geochemical background values, the reference of Martin and Meybeck (1979) was used. Additionally, the contamination factor (C_f_) for seven pollutants (As, Cd, Cu, Cr, Pb, Zn as suggested by Hakanson (1980) and Ni that showed high sediment concentrations) was calculated, along with the degree of contamination for each station (C_d_). For the contamination factor, the same background concentrations as for the I_geo_ index were used. The values of the C_f_ factor and the C_d_ degree correspond to different intensity of contamination (Hakanson 1980).

### Results

The annual fluctuations of the abiotic variables per station and season are given in the Supplementary material (Suppl. material 1)

**Figure 2.**
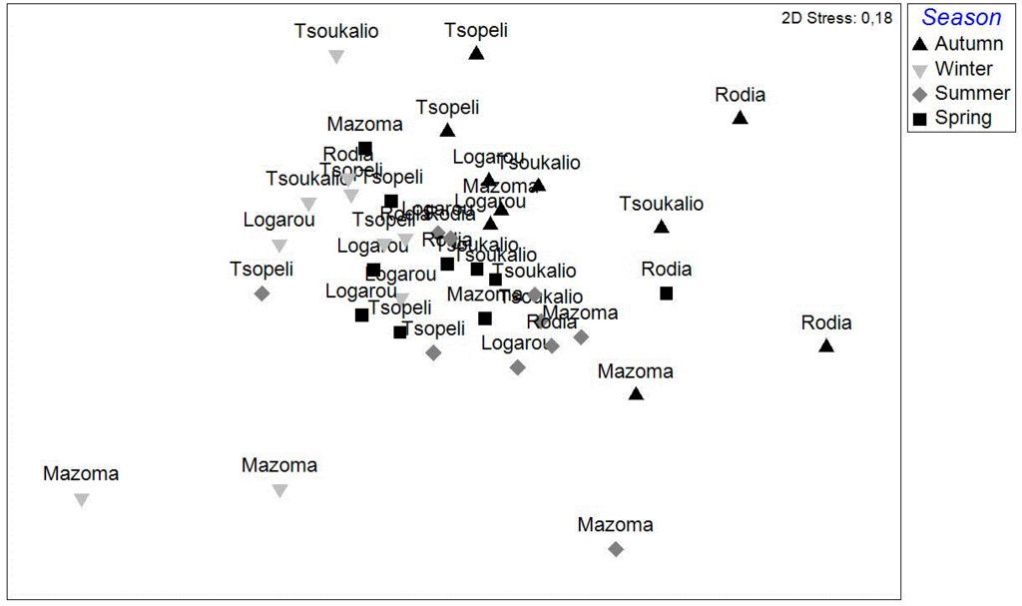
nMDS analysis plot between the stations based on the abiotic data from all the lagoons and sampling seasons.

**Figure 3.**
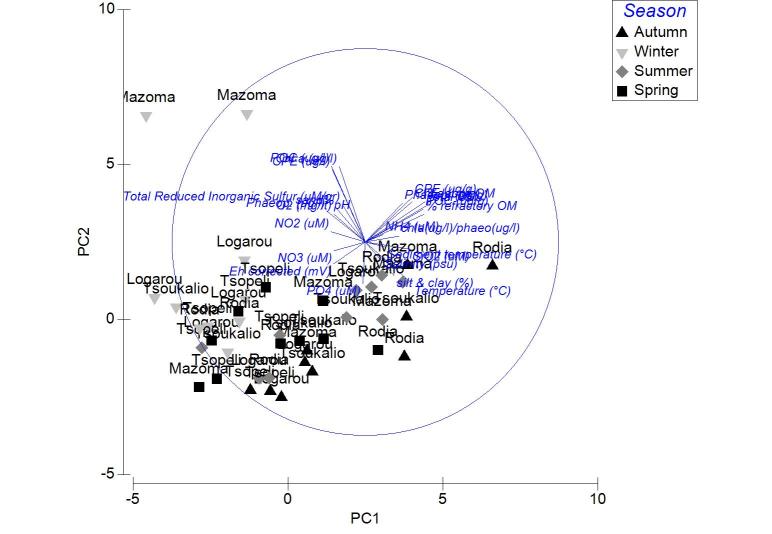
PCA plot of the stations based on the environmental data from all the lagoons and sampling seasons.

The nMDS ordination analysis, applied on the values per station over all seasons, revealed the multivariate similarity patterns based on the values of the abiotic variables (Fig. 2). The PERMANOVA analysis supports clustering of the stations by season (pseudo-F=9.048, p=0.001), by lagoon (pseudo-F=4.018, p=0.001) and by lagoon over season (pseudo-F=2.256, p=0.001). The PCA performed on the same dataset showed that 51.1 % of the total variation was described by the first three PCA axes (λ>1) but no distinguishable eigenvector was observed for any of the abiotic variables tested (Fig. 3).

The results of the nMDS analysis applied on the data per station over single seasons showed no significant grouping between the stations in autumn and spring, while in winter and summer (Fig. 4) there was clear assembly of the stations from the same lagoon (PERMANOVA: pseudo-F=2.949, p<0.05 for winter; pseudo-F=2.384, p<0.05 for summer). The distance from the sea was not found to play any role on the sample clustering.

Applied on the same datasets, the PCA analysis revealed the first two PCA axes (λ>1) identified accounting for the 63.2% of the total variation in the autumn samples. The first principal component was not found to be strongly associated with any of the variables. On the contrary, the second axis was associated (0.509) to the CPE of the sediment (Fig. 5a). For the winter samples the 73% of the variability was explained by the first two axes (λ>1) (Fig. 5b). The first principal component was strongly correlated to the chl-*a* of the water column (-0.50), while the second component was strongly associated with the NO_3_ (-0.507). The 65% of the variability was expressed by the first two components (λ>1) in PCA from the spring stations (Fig. 5c). Nevertheless, only the first one was found to be strongly associated to the following variables: the percentage of silt and clay (0.517) and the percentage of sand in the sediment (-0.517). In the PCA with the data from the summer sampling, only the first two axes were found to fulfill the Kaiser-Guttman criterion and they described the 64.8% of the total variability. No variable was associated to the first principal component, however, the second axis was characterized by the NH_4_ (-0.677) (Fig. 5d).

The nMDS analysis was also applied on data deriving from each lagoon, separately and over all seasons (Fig. 6). The results did show significant grouping of the stations except for Rodia lagoon. In all other lagoons PERMANOVA supported the grouping of stations following a seasonal pattern: pseudo-F=4.952, p<0.05 for Mazoma lagoon; pseudo-F=4.802, p<0.05 for Logarou lagoon; pseudo-F=3.647, p<0.05 for Tsopeli lagoon; pseudo-F=2.839, p<0.05 for Tsoukalio lagoon. The stations did not appear to be grouped according to their distance from the sea in any of the cases described above.

The results of PCA analysis applied on the data from Mazoma lagoon (Fig. 7a), showed that the 70.3% of the total variability was explained by the first two axes, which met the Kaiser-Guttman criterion. However, no variable was strongly associated with any of the components. The same analysis for Logarou lagoon indicated the first three PCA components (λ>1) to represent the 86.7% of the variability (Fig. 7b). Similarly to Mazoma, no variable was strongly associated to any of the three axes. For the data from Tsopeli lagoon, the highest percentage of the total variation was explained by the first two principal components (65.6%). The first component was strongly associated with the phaeophytin in the water column (-0.51), the second one by NO_2_ (-0.524) (Fig. 7c). Similarly, for the data from Tsoukalio lagoon the first two axes (λ>1) accounted for the 67.2% of the variability (Fig. 7d). The NO_3_ was strongly associated (-0.516) to the first component, the second component was strongly related to the ratio of chl-*a*/phaeophytin in the water (-0.683). Finally, for the samples from Rodia, the results of the PCA showed the first two components (λ>1) to represent the 71.3% of the total variability, but no variable was found to be strongly associated (Fig. 7e).

Again, the distance of the stations from the sea, did not appear to be related with any of the vectors identified by the PCA.

All the abiotic variables were plotted against salinity in each lagoon, to give an estimate of the concentration ranges in relation to the freshwater inflows. The linear regression plots of the parameters significantly related to the salinity shifts, are provided in Suppl. material 1. In Mazoma lagoon, the concentration of NH_4_ in the water (R^2^=0.53; p<0.05), phaeophytin (R^2^=0.63; p<0.05) and CPE (R^2^=0.55; p<0.05) in the sediment showed significant increase, by increasing salinity, while the concentration of O_2_ (R^2^=0.77; p<0.05) in the water column appeared to be decreasing with increasing salinity. The same test for Logarou lagoon, indicated significant reduction (R^2^=0.91; p<0.05) of the water POC concentration values as the salinity increased. The picture was different for Tsopeli lagoon, where three environmental factors presented significant changes by the increasing salinity values: temperature in the water column (R^2^=0.73; p<0.05) was augmenting, while the concentration of NO_2_ (R^2^=0.52; p<0.05) and O_2_ (R^2^=0.94; p<0.05) in the water were significantly lowered with increasing salinity. In Tsoukalio lagoon, the concentrations of SiO_2_ (R^2^=0.52; p<0.05) in the water and chl-*a* in the sediments (R^2^=0.50; p<0.05) were higher as salinity was increasing. On the contrary, the values of O_2_ (R^2^=0.70; p<0.05) in the water along with the values of Eh (R^2^=0.52; p<0.05) in the sediments were reducing with the rising of salinity. Finally, the concentration decrement of O_2_ (R^2^=0.85; p<0.05) in the water was the factor that showed significant shifts as the salinity was increasing, in Rodia lagoon.

**Figure 9.**
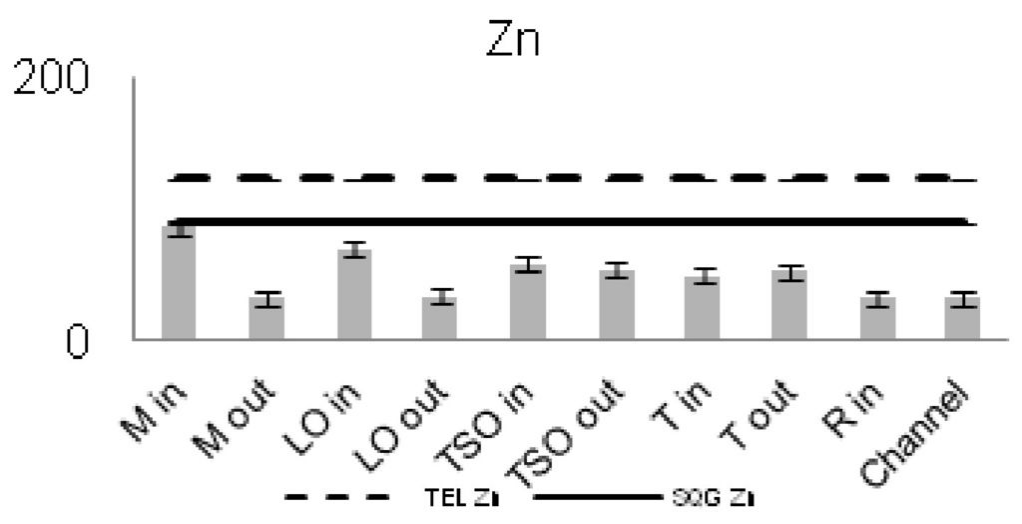
Zn concentrations in the sediment from each station. The dashed line represents the TEL threshold. The continuous line represents the SQG threshold. Abbreviations of the stations as in Fig. 8.

The concentrations of seven of the heavy metals analyzed were compared between the sampling stations (Figs 8, 9). The As concentrations were found to exceed the SQG threshold (3 ppm) in all the stations except from the outer station of Logarou (2.38 ppm) but they never transcended the TEL limit (5.9 ppm). Similarly, the concentration of Cu was found to be above the SQG level (25 ppm) and, in the cases of the inner stations of Mazoma (45.87 ppm) and Logarou (36.51 ppm), even higher than the TEL level (35.7 ppm). The lowest values of Cu were recorded to the outer station of Rodia lagoon (14.48 ppm). The results were different for Cr and Ni. The concentrations of these elements were found to be high in all the stations and exceeding the levels of both SQG (SQG_Cr_=25; SQG_Ni_=20) and TEL (TEL_Cr_=37.3; TEL_Ni_=18).

The results of the I_geo_ index classified most of the stations to the zero class of the uncontaminated sediments (Table 2). The inner station of Mazoma was the exception in this pattern, as it was ranked to class one which includes the level of of the uncontaminated to moderately contaminated sediments, based on the Cd and Pb and to class two, which includes the moderately contaminated sediments based on the Cr and Ni. However, the index did not rank sediments of this station as contaminated by As, Cu and Zn. The sediments were classified to low or moderate Cr contamination for all the stations and to low and moderate Ni contamination for most of the stations. Overall, the index was high for Cr and Ni and low for Zn and Pb. The order of the elements as ranked by the I_geo_ index values was Cr>Ni>Cd>Cu>As>Zn>Pb.

Similar results were derived from the calculation of the contamination factor (C_f_) (Fig. 10). The factor for the inner station of Mazoma was found to be above the level of low contamination for the Cd, Cu, and Pb and above the moderate contamination level for Cr and Ni. The values were exceeding the low contamination threshold for Cd in both of the Tsopeli stations and for Cu and Pb in the sediment of the inner station from Logarou lagoon. The values of Cr and Ni were over the low level of contamination in all the sediment samples.

The contamination degree was calculated for all seven metals (Fig. 11a) and the mean contamination degree was estimated for all the stations (Fig. 11b). Results of the index presented only three stations under the level of low contamination: the outer stations of Mazoma and Logarou and the inner station of Rodia. According to the average values of the C_d_ (mC_d_) the stations that were ranked as with the highest contamination for all the contaminants were the inner stations of Mazoma and Logarou. The elements with the highest C_d_ values were Cr an Ni in the sediments over all stations. The order of the contaminants following the degree of contamination in the sediments were Cr>Ni>Cd>Cu>As>Zn>Pb.

### Discussion

Overall, no evidence of intense disturbance was observed in patterns derived by the abiotic variables in the lagoons under study. Salinity ranges were negligible between the inner and the outer parts of the lagoons in all seasons. The annual pattern, however, was characterized by high salinity values in the summer and autumn and low in winter and spring. Salinity values follow a pattern similar to those described in the same lagoons from older studies (e.g. Kormas et al. 2001, Katselis et al. 2013), but also in other lagoons from Greece (Kevrekidis 2004, Orfanidis et al. 2005) and the Mediterranean Sea (De Casabianca et al. 1997, Vignes et al. 2009). However, salinity levels in Tsoukalio and Rodia were found much lower than all the other lagoons (5.6-20 psu in Tsoukalio; 4.7-23.4 psu in Rodia). Kormas et al. (2001) have reported higher salinity levels, ranging between 13-28.9 psu in Tsoukalio and 11.6-25 psu in Rodia. Moreover, Nicοlaidou et al. (2005) mentioned salinity ranges between 14.0-36.5 psu for Tsoukalio and 5.0-35.0 psu for Rodia. The low levels of salinity measured in the two lagoons during the study are comparable to the ranges referenced from the Evros Delta (4.0-25.0 psu; Nicοlaidou et al. 2005), and are considered as indicative of increased freshwater inflows. Furthermore, the salinity levels in Tsopeli (14.6-42.1 psu) were found to have a wider range than the ones remarked in the study of Nicοlaidou et al. (2005) (21-38 psu) and the paper of Reizopoulou et al. (1996) (21-35 psu).

The oxygen concentration shifts during the year, were normal for all lagoonal systems studied. Lower concentrations were measured during summer (1.4 - 6.32 mg/l) and autumn (3.66 - 6.45 mg/l), whereas higher values were observed in winter (7 - 9.43 mg/l) and spring (6.85 - 11.7 mg/l). A similar pattern has been observed in Monolimni lagoon (Kevrekidis 2004). Older studies in the lagoons of Amvrakikos reveal high values during autumn (10.4 - 10.7 mg/l) and lower ones (6 - 9.2 mg/l) during the rest of the year (Kormas et al. 2001), but the ranges were generally in accordance with the measurements reported from previous studies in the lagoons (2.8 - 12.1 mg/l) (Reizopoulou and Nicolaidou 2004). The oxygen concentration fluctuations are influenced by the salinity and temperature ranges and by the biological activity (Best et al. 2007). The annual DO pattern observed in the lagoons of Amvrakikos could be explained by the salinity and temperature annual profiles, as increased levels of temperature and salinity inhibit solubility of the dissolved oxygen (Best et al. 2007).

The nMDS plots and PERMANOVA analysis for all stations and over all sampling seasons revealed significant differentiation between the lagoons, between the seasons and also between the lagoons and over the seasons. However, the location of the stations within each lagoon does not appear to be an important factor. This could be attributed to the physicochemical characteristics of each lagoon, as the PCA analysis showed no variable playing a predominant role in the pattern of variation among stations. This pattern seems to change when the data are treated per season. Although no specific grouping of stations was observed during autumn, the concentration of CPE in the sediments seemed to influence their arrangement along the PCA axes. Overall, the CPE levels in the sediment were high. However, in the case of Mazoma, the stations were found to have higher concentrations, which were increasing with the increasing salinity. The percentage of chl-*a* in autumn was about 40%, suggesting that although there was continuous input of primary organic matter in the sediment, it was mostly consisted of accumulated chl-*a* (Pusceddu et al. 1999, Mirto et al. 2004, Sestanovic et al. 2009).

In Mazoma lagoon, the concentration of NH_4_ was also found to increase with the increasing salinity, being highest during summer. This response is anticipated when the degradation of the organic matter in the sediment takes place (Bonanni et al. 1992, Diamantopoulou et al. 2008). However, the summer levels were extremely high in respect to the ones reported from other Mediterranean lagoons (Orfanidis et al. 2005, Cobelo-García et al. 2012, Guerra et al. 2013). The degradation of organic matter in the summer, is also supported by the significant decrease of the oxygen concentration in the water column with the increasing salinity levels. Increased NH_4_ levels are also noticed during hypoxic events (Wu 2002), however the oxygen concentration (4.76 - 9.43 mg/l) was measured to be within the natural ranges (4.8-6.8 mg/l) (Nicοlaidou et al. 2005).

Only the POC concentration in the water column was found to be significantly correlated with the salinity change in Logarou lagoon. The highest concentrations were noticed in winter and they seem to follow the elevated levels of chl-*a* in the water column, over the same period, suggesting phytoplanktonic origin of POC.

In Tsopeli lagoon, the PCA analysis pointed out the concentration of phaeophytin and NO_2_ in the water as the most important factors for the stations ordination. The NO_2_ concentrations were also found to be increasing with the declining salinity levels, indicating inflows through the freshwater. The oxygen concentration annual shifts were following the same pattern as the NO_2_. Oxygen concentration presented the lowest values in this lagoon during summer and autumn, however this seems to be natural for Tsopeli as such a decline has been observed before (Reizopoulou and Nicolaidou 2004).

For Tsoukalio lagoon, the PCA analysis showed that the NO_3_ concentration and the ratio chl-*a*/phaeophytin in the water column were playing an important role on the station pattern throughout the year. The chl-*a*/phaeophytin ratio takes its highest values in autumn. Although the CPE in the water was not high, the percentage of chl-*a* was assessed to reach over 90% of the CPE against the phaeopigment content in autumn, dramatically dropped under 50% in winter and even lower than 30% in spring (to the outer stations). Such a decline of chl-*a* concentrations in the water column, along with a concurrent increase of phaopigments, which are considered as chl-*a* degradation products, is often attributed to intense zooplankton grazing activity (Taguchi et al. 1993).

Similarly to Logarou, fluctuations of the abiotic factors in Rodia seemed to have lower ranges. Oxygen concentration was the only factor found to change significantly in the lagoon, with the increasing salinity levels. The lowest values were measured in the lagoon during summer, nevertheless they were not found to be out of the ranges described for the lagoon by previous studies (Kormas et al. 2001).

The concentrations of heavy metals were higher than the SQG threshold in many cases, but lower than the TEL threshold for the majority of the elements. However, the concentrations of Cr and Ni were exceeding both of the limits in all the stations. The same elements were classified to the class one of the index for all the lagoons and they were surpassing the level of low sediment contamination of the C_d_ index. The mean contamination factor was exceeding the lower limit of contamination for all the stations, but none of them was over the threshold of moderate contamination. However, the concentrations of these elements in the lagoonal sediments were found in lower levels than those reported by Christophoridis et al. 2007 in the same lagoons. Karageorgis (2007) reported also high values of Cr and Ni concentration in the sediments of Tsopeli, Rodia, Tsoukalio and Logarou lagoons. The author argued that the elements were contained in the soil minerals of Arachthos River and they are transported in the lagoons through the river inflows.

No evidence of severe disturbance was detected in the lagoons of Amvrakikos. The annual fluctuations of the parameters were following the profiles reported before in the area, suggesting that the physicochemical functions in the lagoons do not suffer any major impact. The annual oxygen fluctuations were found to be significant in most of the lagoons studied. Oxygen concentration values were higher during winter and spring and were severely dropping in summer and autumn, following the pattern already described by other authors for the Mediterranean lagoons (e.g. Sfriso et al. 1992, De Casabianca et al. 1997). However, there were no indications of dystrophic crises events in the samples collected from the Amvrakikos Gulf lagoons and the analysis of the heavy metal concentrations showed no evidence of severe contamination. The lagoons are exploited for their high fish production. Therefore, their optimal environmental conditions are important not only for the welfare of the ecosystem, but also for the prosperity of the local community.

## Supplementary Material

Supplementary material 1 Supplementary 1 Data type: analyses results
File: oo_99063.docx K. Vasileiadou, C. Pavloudi, I. Kalantzi, E. Apostolaki, G. Chatzigeorgiou, E. Chatzinikolaou, E. Pafilis, N. Papageorgiou, L. Fanini, S. Konstas, N. Fragopoulou, C. Arvanitidis

## Figures and Tables

**Figure 4a.**
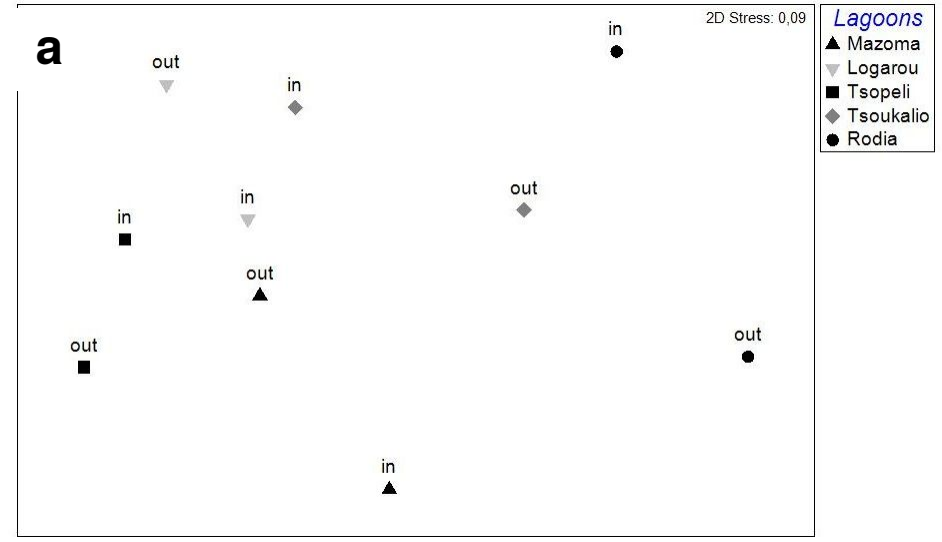
Autumn

**Figure 4b.**
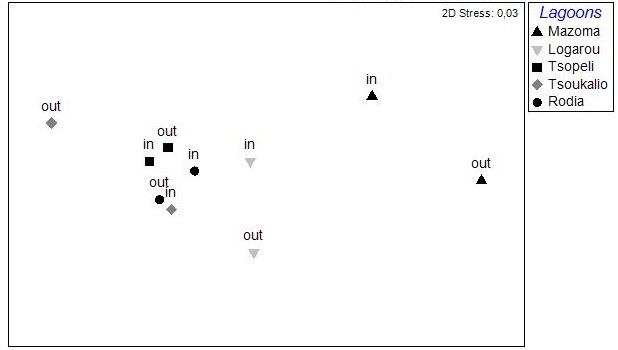
Winter

**Figure 4c.**
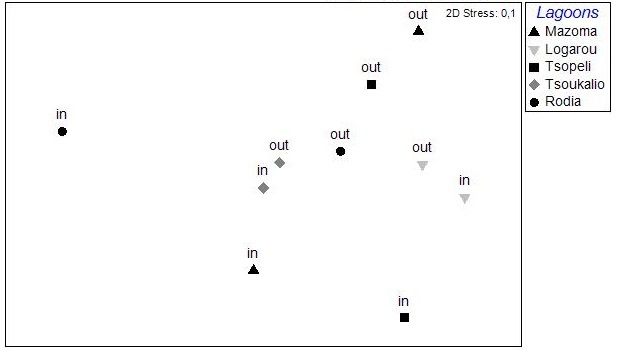
Spring

**Figure 4d.**
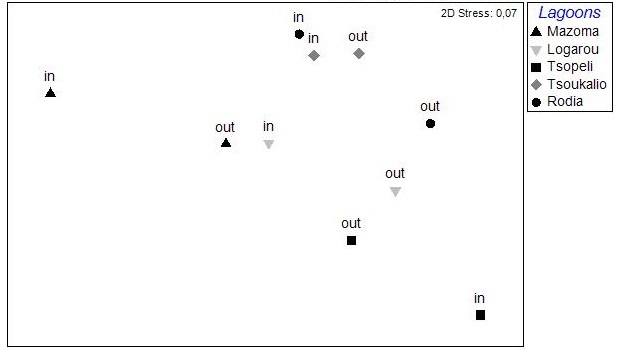
Summer

**Figure 5a.**
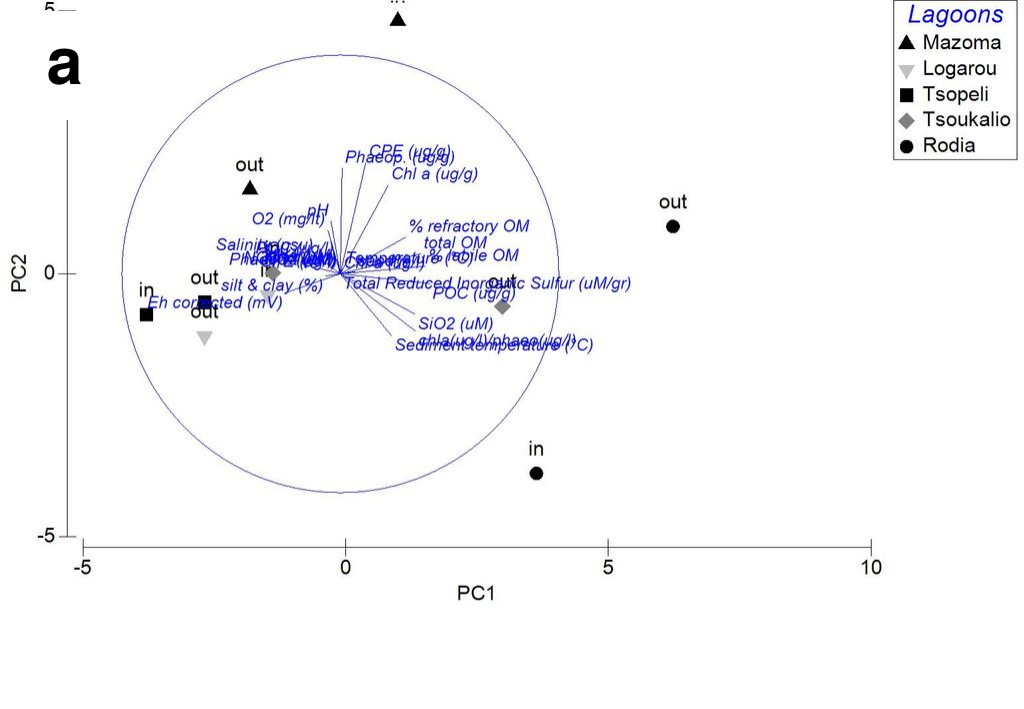
Autumn

**Figure 5b.**
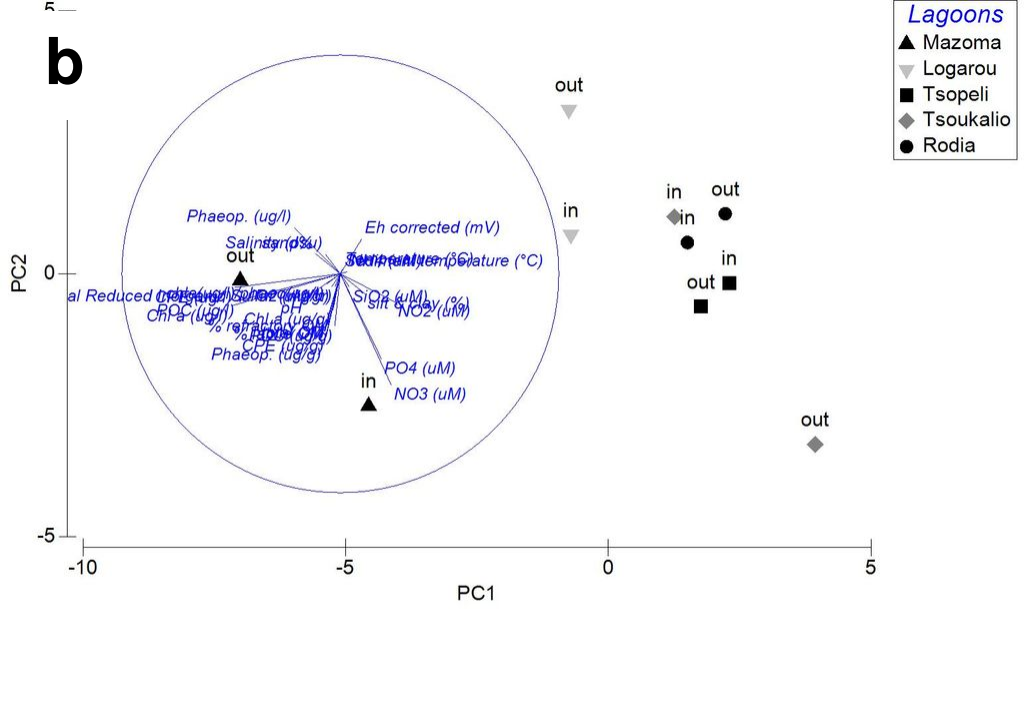
Winter

**Figure 5c.**
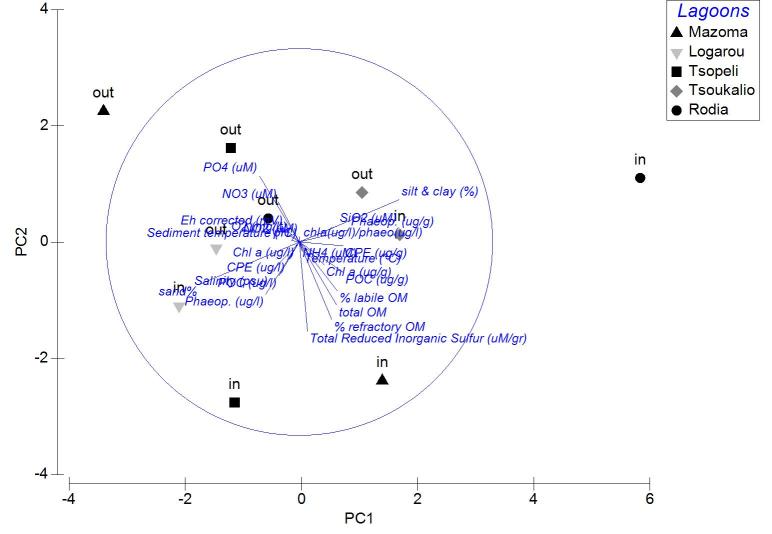
Spring

**Figure 5d.**
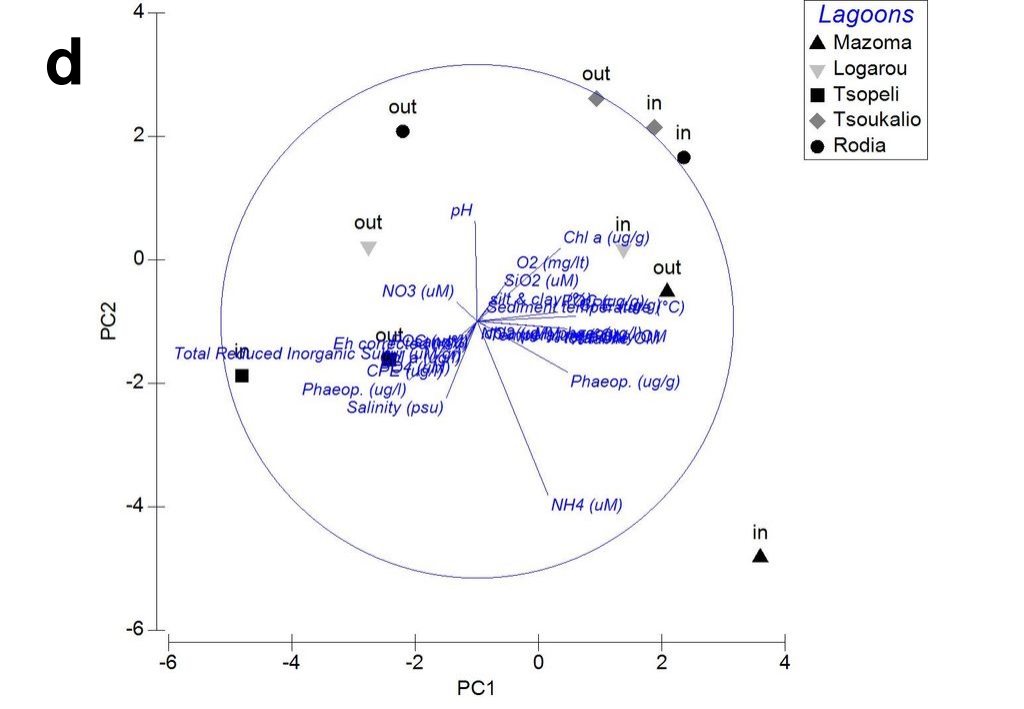
Summer

**Figure 6a.**
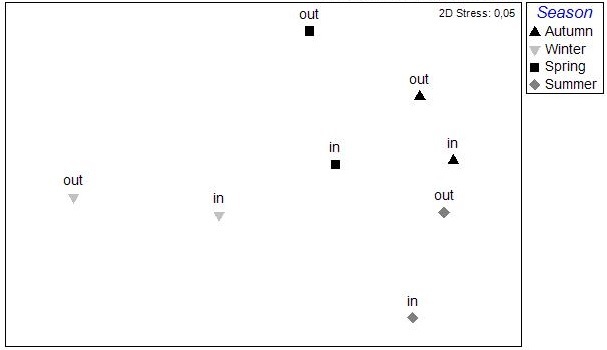
Mazoma

**Figure 6b.**
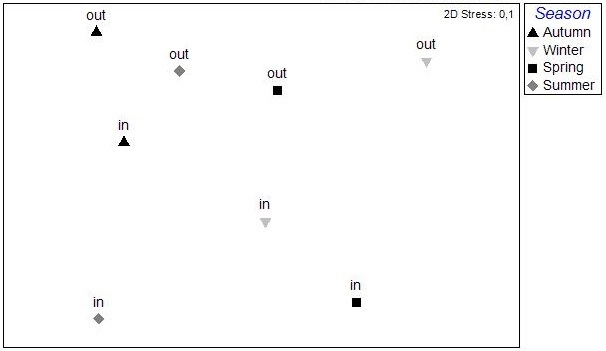
Logarou

**Figure 6c.**
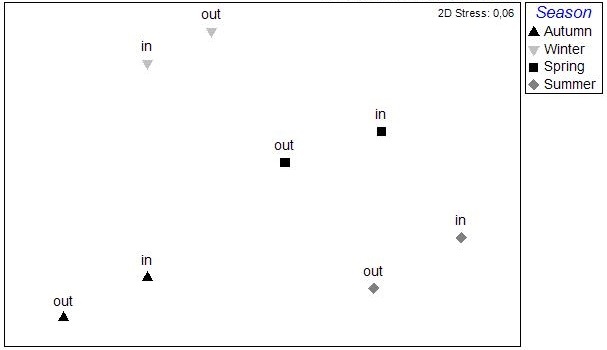
Tsopeli

**Figure 6d.**
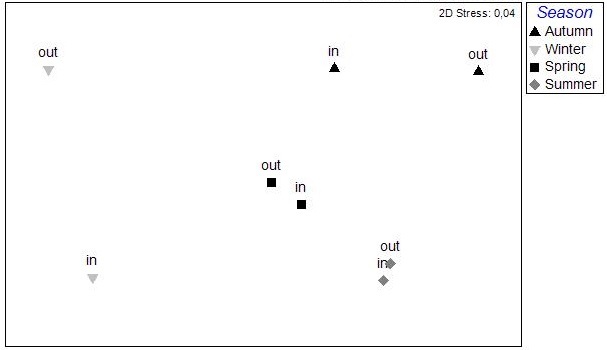
Tsoukalio

**Figure 6e.**
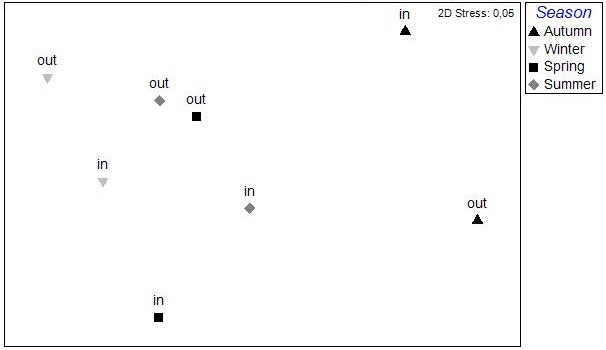
Rodia

**Figure 7a.**
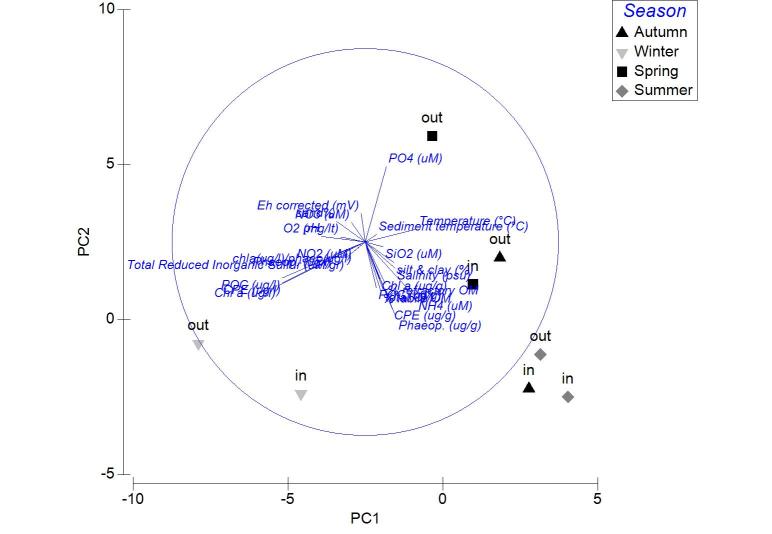
Mazoma

**Figure 7b.**
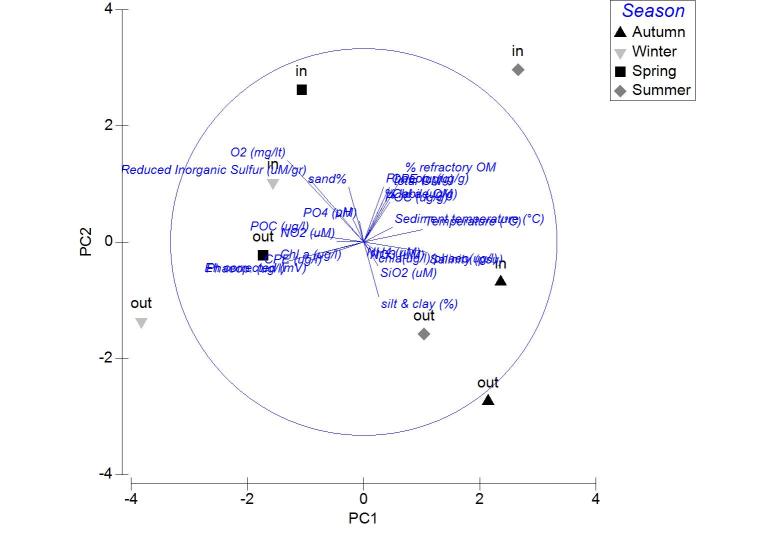
Logarou

**Figure 7c.**
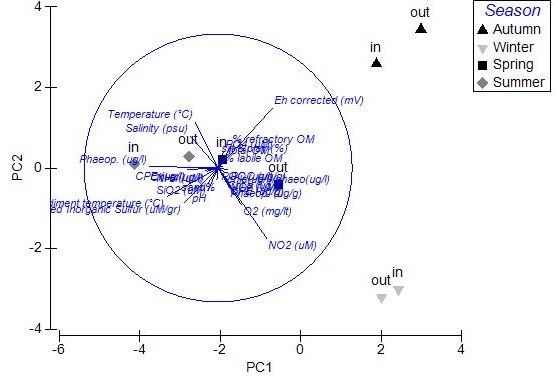
Tsopeli

**Figure 7d.**
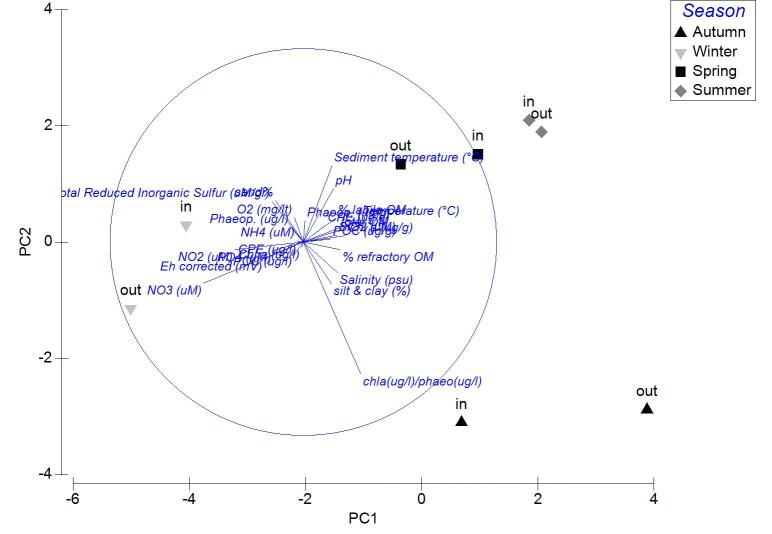
Tsoukalio

**Figure 7e.**
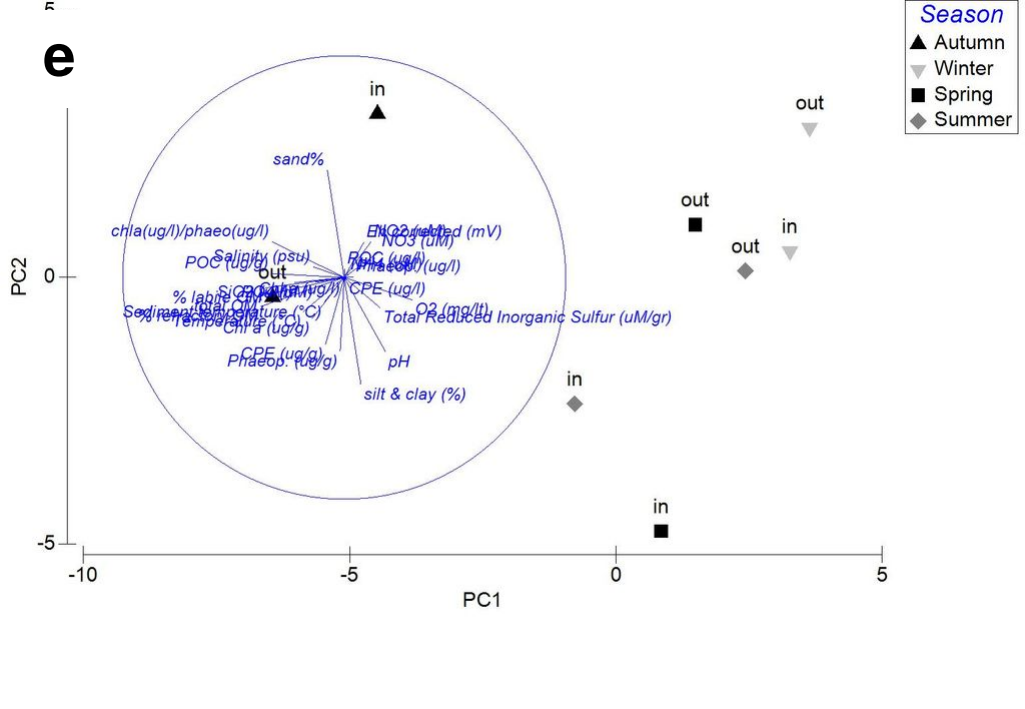
Rodia

**Figure 8a.**
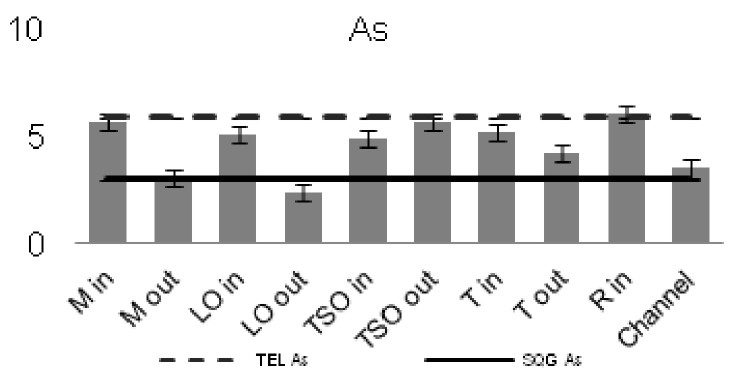


**Figure 8b.**
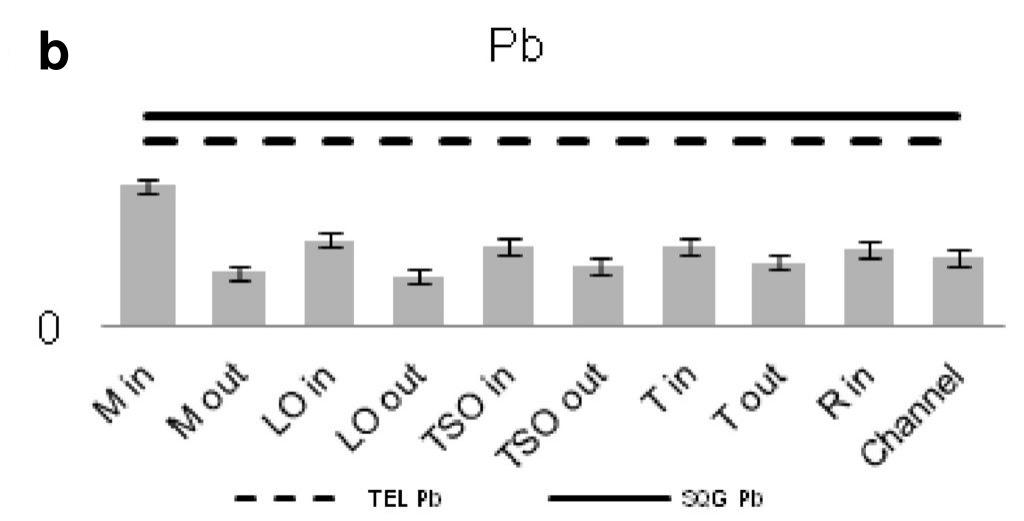


**Figure 8c.**
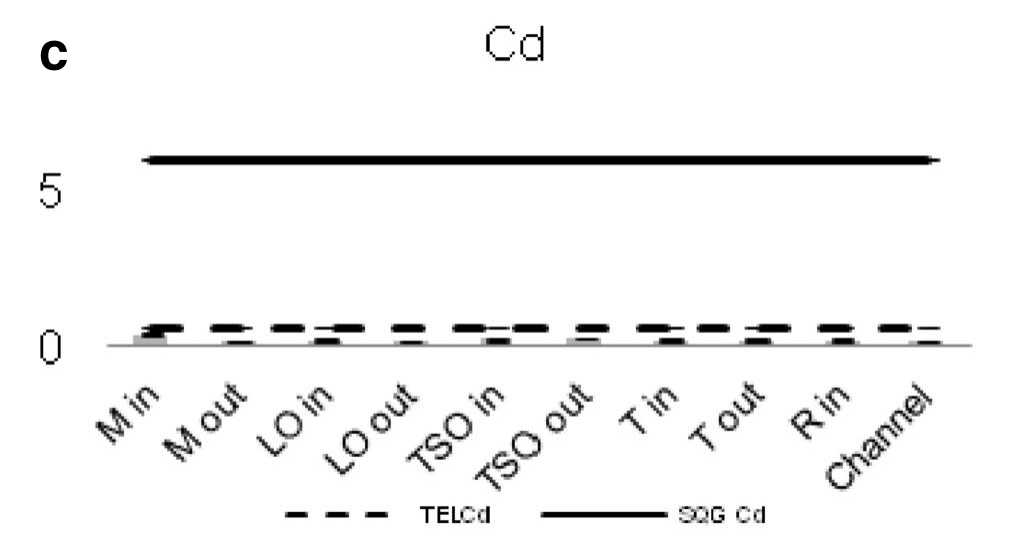


**Figure 8d.**
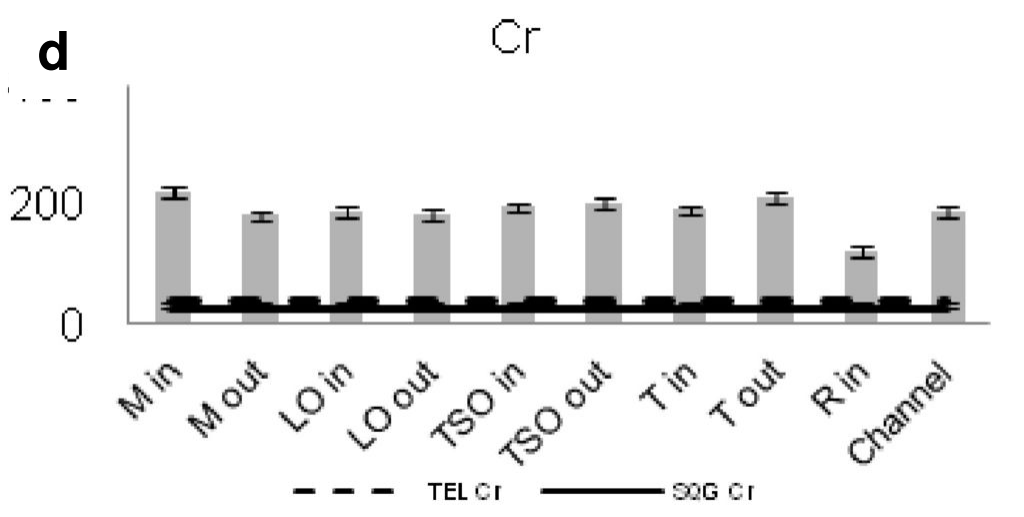


**Figure 8e.**
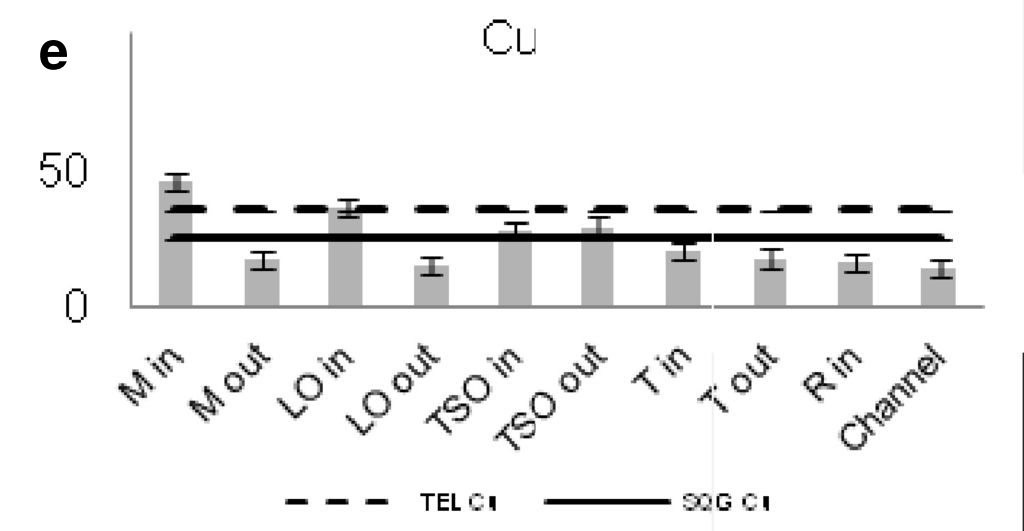


**Figure 8f.**
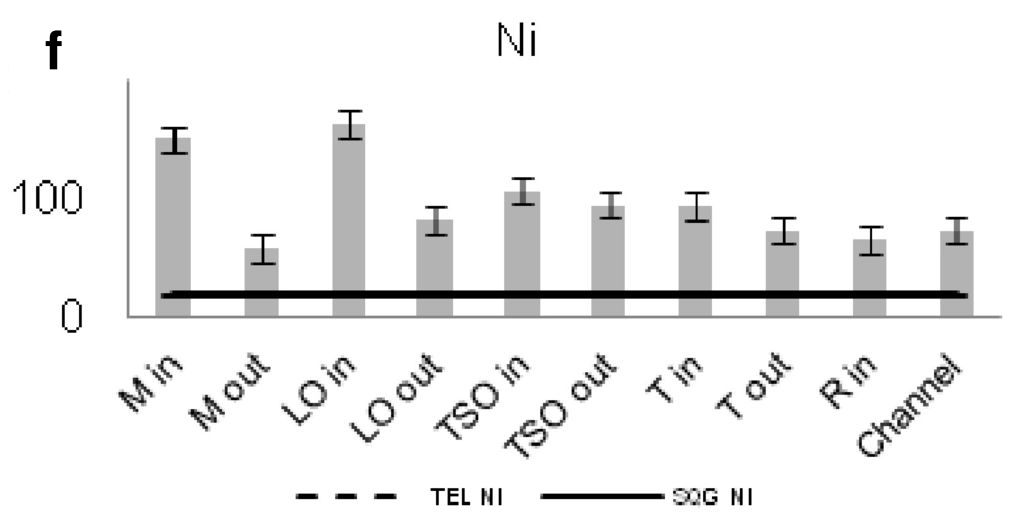


**Figure 10a.**
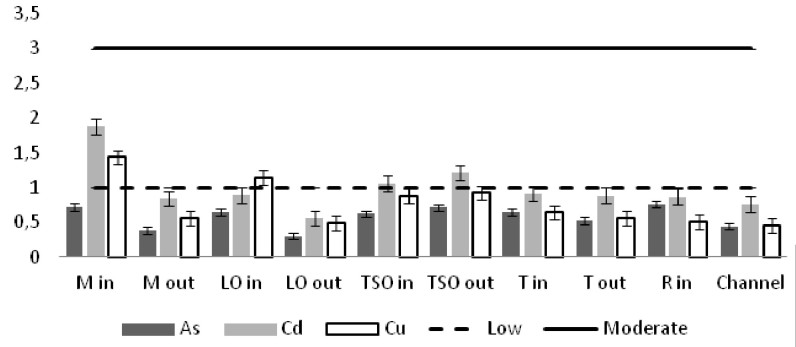
index values for the As, Cd, and Cu

**Figure 10b.**
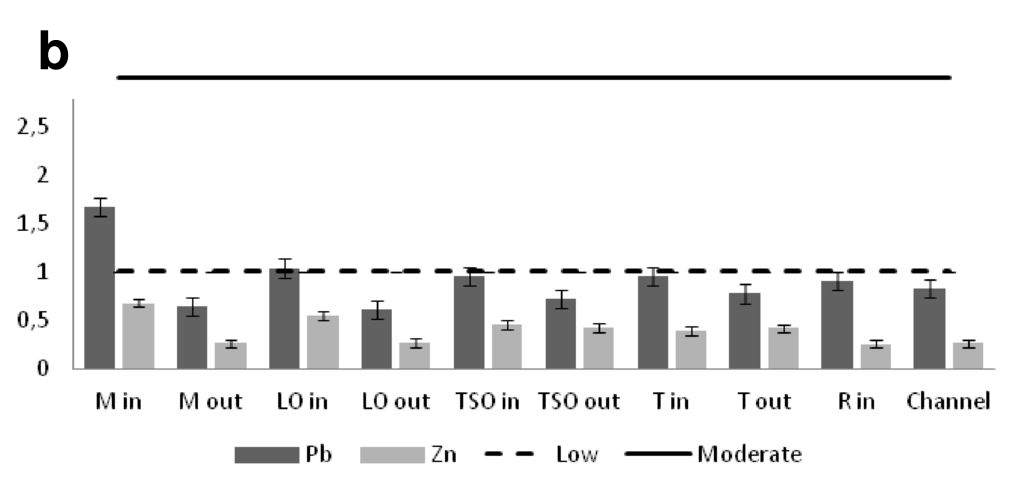
index values for Pb and Zn

**Figure 10c.**
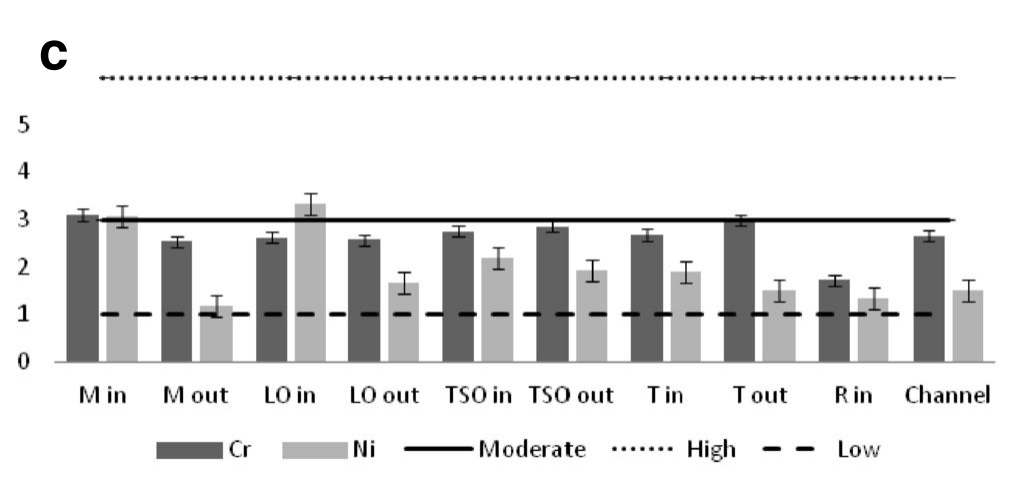
index values for Cr and Ni

**Figure 11a.**
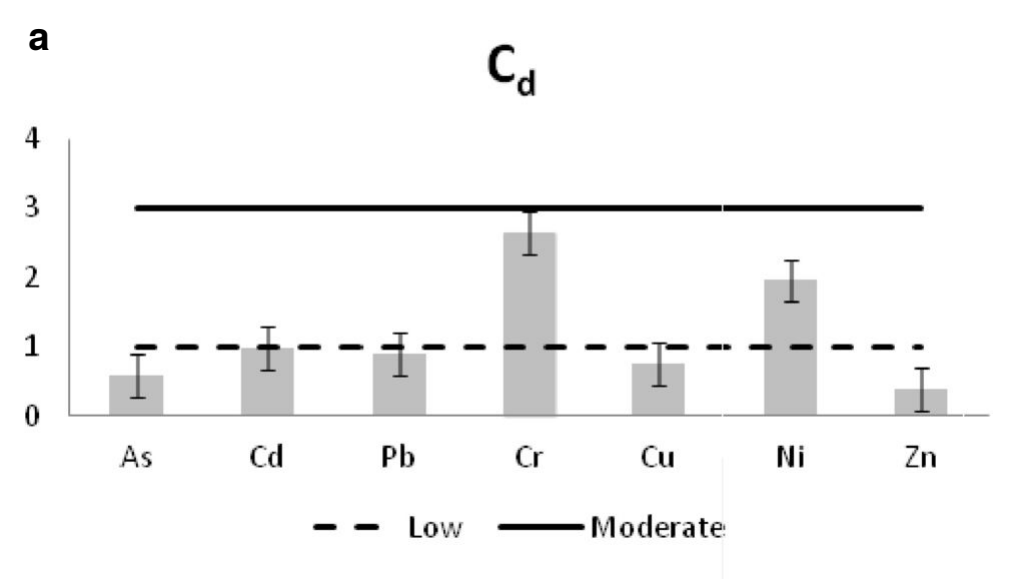
The Contamination degree values for each element

**Figure 11b.**
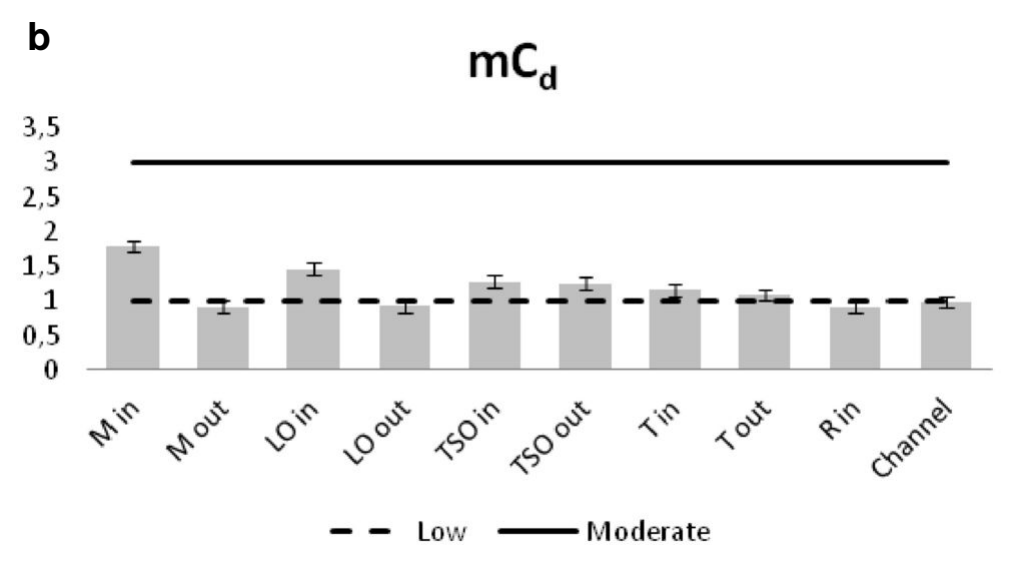
The mean Contamination degree values per station

**Table 1.** The abiotic variables measured from the lagoons of Amvrakikos. The parenthesis include the units of each parameter.

Water column	Salinity
	Temperature (°C)
	Ammonium - NH_4_ (uM)
	Phosphate - PO_4_ (uM)
	Nitrate - NO_3_ (uM)
	Nitrite - NO_2_ (uM)
	Silicon dioxide - SiO_2_ (uM)
	Chlorophyl-*a* (ug/l)
	Phaeopigments (ug/l)
	Chloroplastic Pigment Equivalents (ug/l)
	Particulate Organic Carbon (ug/l)
	pH
	Oxygen - O_2_ (mg/lt)
Sediment	Temperature (°C)
	Total Reduced Inorganic Sulfur (TRIS) (uM)
	Chlorophyl-*a* (ug/g)
	Phaeopigments (ug/g)
	Chloroplastic Pigment Equivalents (ug/g)
	Particulate Organic Carbon (ug/g)
	Labile Organic Matter (%)
	Refractory Organic Matter (%)
	Total Organic Matter
	Redox potential - Eh (mV)
	Silt and Clay (%)
	Sand (%)

**Table 2.** The I_geo_ index values and the enrichment class of each element per station.

		M in	M out	LO in	LO out	TSO in	TSO out	T in	T out	R in	Channel	Average
**I_geo_**	As	-1.06	-1.95	-1.22	-2.32	-1.27	-1.08	-1.2	-1.5	-0.98	-1.76	-1.43
	Cd	0.32	-0.83	-0.75	-1.43	-0.5	-0.31	-0.72	-0.77	-0.8	-0.98	-0.68
	Pb	0.15	-1.21	-0.53	-1.3	-0.65	-1.06	-0.65	-0.95	-0.72	-0.86	-0.78
	Cr	1.05	0.76	0.81	0.78	0.88	0.92	0.84	0.99	0.2	0.82	0.81
	Cu	-0.07	-1.44	-0.39	-1.62	-0.77	-0.7	-1.23	-1.42	-1.55	-1.73	-1.09
	Ni	1.03	-0.35	1.15	0.15	0.54	0.36	0.35	-0.01	-0.17	-0.001	0.30
	Zn	-1.14	-2.53	-1.46	-2.49	-1.7	-1.82	-1.94	-1.85	-2.55	-2.53	-2.00
**Class**	As	0	0	0	0	0	0	0	0	0	0	0
	Cd	1	0	0	0	0	0	0	0	0	0	0
	Pb	1	0	0	0	0	0	0	0	0	0	0
	Cr	2	1	1	1	1	1	1	1	1	1	1
	Cu	0	0	0	0	0	0	0	0	0	0	0
	Ni	2	0	2	1	1	1	1	0	0	0	1
	Zn	0	0	0	0	0	0	0	0	0	0	0
