# Supplementary material for: Environmental variability and heavy metal concentrations from five lagoons in the Ionian Sea (Amvrakikos Gulf, W Greece)
Source: Biodivers Data J. 2016 Nov 1;(4):e8233. doi: 10.3897/BDJ.4.e8233 (PMC5136647; doi:10.3897/BDJ.4.e8233)
Supplement: Supplementary material 1 — Supplementary 1 [file biodiversity_data_journal-4-e8233-s001.docx]

**Supplementary material**

Values of the abiotic variables per station and season

| summer | summer |
| --- | --- |
| summer | summer |
| summer | summer |
| summer | summer |
| summer | summer |
| summer | summer |
| summer | summer |
| **summer** | summer |
| summer | summer |
| summer  summer | summer |

Percentage of chl-*a* and Phaeopigments in the CPE

Linear regression plots

| 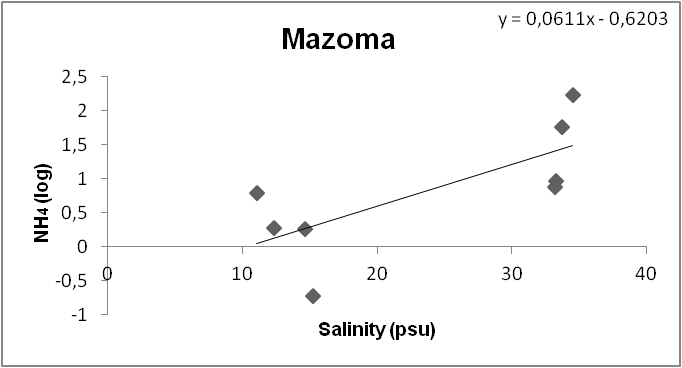 | 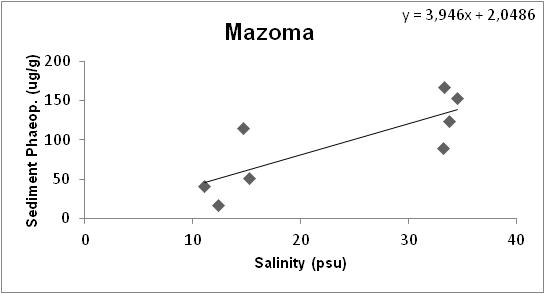 |
| --- | --- |
| 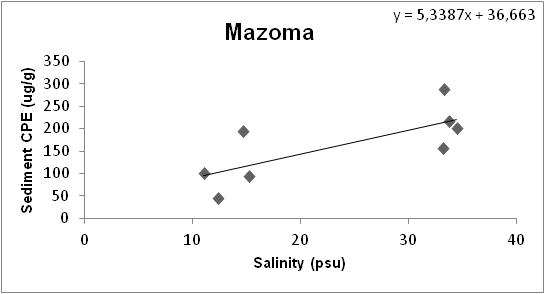 | 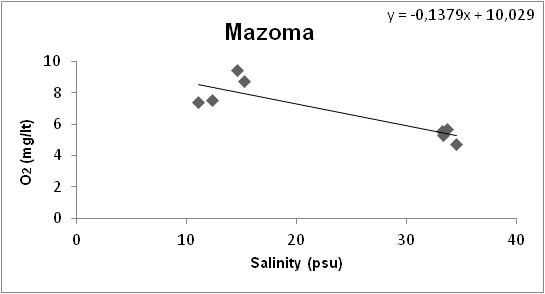 |

| 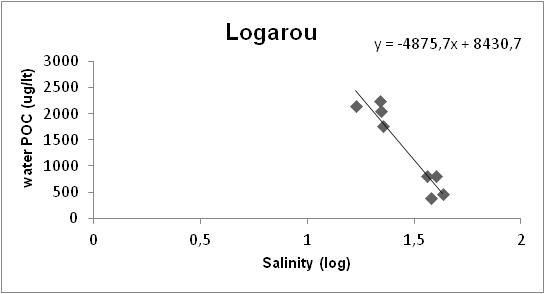 | 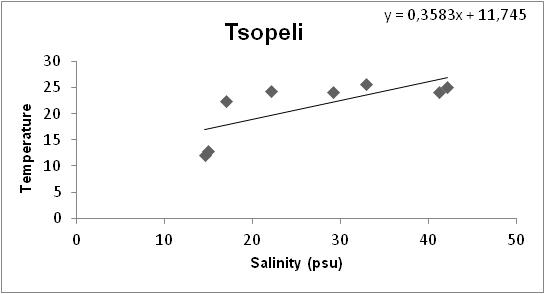 |
| --- | --- |
| 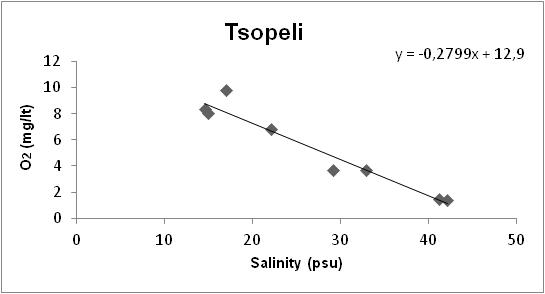 | 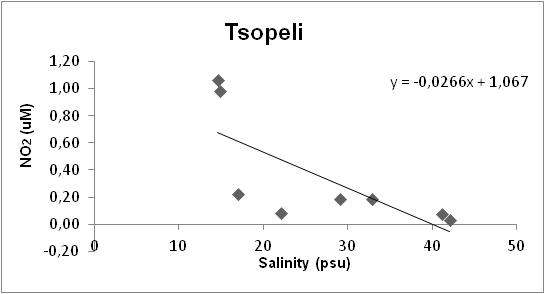 |
| 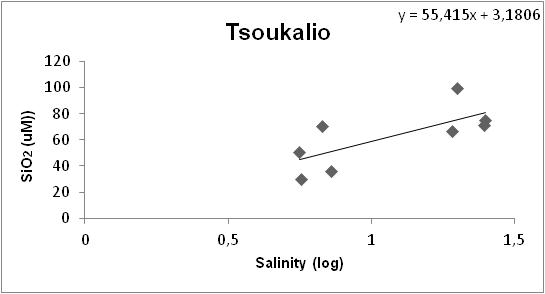 | 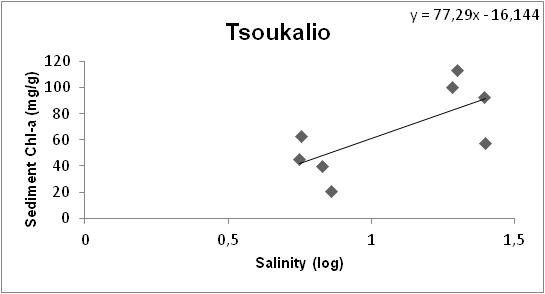 |
| 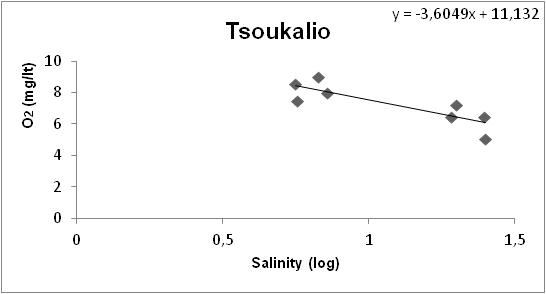 | 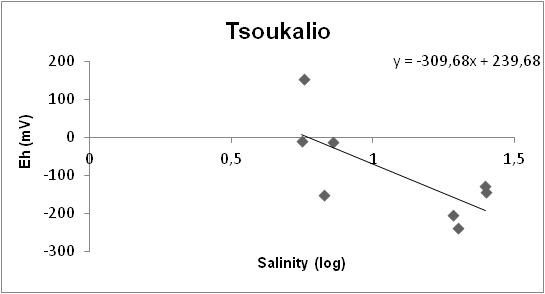 |
| 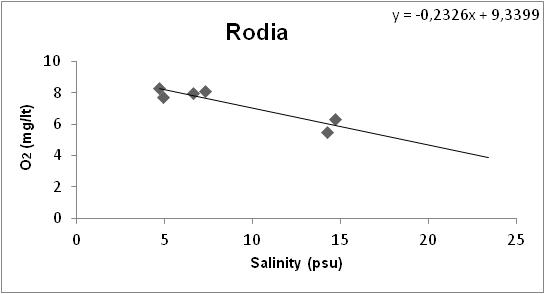 |  |

PCA Results

| **I. PCA eigenvectors for all stations and seasons** | | | | | |
| --- | --- | --- | --- | --- | --- |
| **Variable** | **PC1** | **PC2** | **PC3** | **PC4** | **PC5** |
| Salinity (psu) | 0.085 | -0.076 | -0.316 | -0.368 | -0.236 |
| NH_4_ (uM) | 0.093 | 0.038 | -0.139 | -0.025 | -0.084 |
| PO_4_ (uM) | -0.013 | -0.216 | 0.132 | -0.031 | 0.15 |
| NO_3_ (uM) | -0.162 | -0.046 | 0.348 | 0.111 | 0.052 |
| NO_2_ (uM) | -0.178 | 0.053 | 0.408 | 0.071 | 0.001 |
| SiO_2_ (uM) | 0.234 | -0.037 | -0.011 | 0.139 | 0.345 |
| Water chl-*a* (ug/l) | -0.134 | 0.39 | -0.117 | -0.132 | -0.065 |
| Water Phaeop. (ug/l) | -0.198 | 0.159 | -0.262 | -0.112 | 0.082 |
| Water CPE (ug/l) | -0.171 | 0.374 | -0.175 | -0.144 | -0.031 |
| Water POC (ug/l) | -0.175 | 0.391 | -0.03 | -0.138 | -0.063 |
| TRIS (uM/gr) | -0.118 | 0.194 | -0.092 | 0.279 | 0.18 |
| Sediment chl-*a* (ug/g) | 0.244 | 0.209 | 0.095 | 0.101 | -0.174 |
| Sediment Phaeop. (ug/g) | 0.193 | 0.201 | 0.029 | 0.153 | -0.365 |
| Sediment CPE (ug/g) | 0.244 | 0.232 | 0.066 | 0.147 | -0.317 |
| Sediment POC (ug/g) | 0.3 | 0.169 | 0.204 | -0.042 | 0.203 |
| % labile OM | 0.31 | 0.209 | 0.129 | -0.043 | 0.187 |
| % refractory OM | 0.304 | 0.143 | 0.101 | -0.156 | 0.052 |
| total OM | 0.321 | 0.19 | 0.123 | -0.092 | 0.139 |
| Temperature (°C) | 0.242 | -0.215 | -0.302 | -0.032 | 0.034 |
| Sediment temperature (°C) | 0.103 | -0.026 | -0.351 | 0.261 | 0.345 |
| pH | -0.069 | 0.149 | -0.145 | 0.383 | 0.022 |
| O_2_ (mg/lt) | -0.163 | 0.145 | 0.115 | 0.388 | 0 |
| Eh (mV) | -0.166 | -0.114 | 0.305 | -0.266 | -0.113 |
| silt & clay (%) | 0.15 | -0.175 | -0.025 | 0.201 | -0.341 |
| sand% | -0.15 | 0.175 | 0.025 | -0.201 | 0.341 |
| water chl-*a*/phaeop. (ug/l) | 0.173 | 0.03 | 0.11 | -0.263 | 0.131 |

| Eigenvalues | |  |  |
| --- | --- | --- | --- |
| PC | Eigenvalues | %Variation | Cum.%Variation |
| 1 | 6,68 | 25,7 | 25,7 |
| 2 | 3,85 | 14,8 | 40,5 |
| 3 | 2,74 | 10,5 | 51,1 |
| 4 | 2,53 | 9,7 | 60,8 |
| 5 | 2,07 | 8 | 68,7 |

| **II. PCA eigenvectors for Autumn** | | | | | |
| --- | --- | --- | --- | --- | --- |
| **Variable** | **PC1** | **PC2** | **PC3** | **PC4** | **PC5** |
| Salinity (psu) | -0,112 | 0,083 | -0,031 | 0,211 | -0,01 |
| NH_4_ (uM) | -0,008 | 0,035 | 0,018 | 0,001 | 0,037 |
| PO_4_ (uM) | -0,002 | 0,016 | 0,357 | -0,147 | -0,25 |
| NO_3_ (uM) | -0,102 | 0,025 | 0,022 | -0,197 | -0,029 |
| NO_2_ (uM) | -0,008 | 0,017 | 0,11 | 0,017 | 0,039 |
| SiO_2_ (uM) | 0,342 | -0,184 | -0,101 | -0,09 | 0,116 |
| Water chl-*a* (ug/l) | 0,003 | 0,002 | -0,009 | -0,038 | -0,036 |
| Water Phaeop. (ug/l) | -0,017 | 0,015 | 0,026 | 0,002 | -0,007 |
| Water CPE (ug/l) | -0,002 | 0,006 | 0 | -0,032 | -0,032 |
| Water POC (ug/l) | -0,017 | 0,066 | 0,009 | 0,006 | 0,05 |
| TRIS (uM/gr) | 0 | 0 | 0 | 0 | 0 |
| Sediment chl-*a* (ug/g) | 0,22 | 0,406 | -0,171 | -0,012 | -0,177 |
| Sediment Phaeop. (ug/g) | 0,008 | 0,483 | 0,026 | -0,009 | 0,352 |
| Sediment CPE (ug/g) | 0,117 | 0,509 | -0,07 | -0,012 | 0,131 |
| Sediment POC (ug/g) | 0,41 | -0,045 | 0,193 | -0,086 | 0,195 |
| % labile OM | 0,39 | 0,035 | 0,205 | 0,037 | 0,073 |
| % refractory OM | 0,3 | 0,168 | 0,241 | 0,038 | -0,391 |
| total OM | 0,369 | 0,092 | 0,229 | 0,039 | -0,118 |
| Temperature (°C) | 0,01 | 0,022 | 0,028 | 0,026 | 0,037 |
| Sediment temperature (°C) | 0,235 | -0,284 | -0,186 | 0,439 | -0,118 |
| pH | -0,043 | 0,241 | -0,32 | 0,162 | -0,64 |
| O_2_ (mg/lt) | -0,057 | 0,199 | -0,026 | -0,485 | -0,016 |
| Eh (mV) | -0,251 | -0,087 | 0,535 | -0,199 | -0,291 |
| silt & clay (%) | -0,066 | -0,009 | -0,234 | -0,251 | -0,04 |
| sand% | 0,066 | 0,009 | 0,234 | 0,251 | 0,04 |
| water chl-*a*/phaeop. (ug/l) | 0,345 | -0,262 | -0,297 | -0,503 | -0,142 |

| Eigenvalues | |  |  |
| --- | --- | --- | --- |
| PC | Eigenvalues | %Variation | Cum.%Variation |
| 1 | 10,9 | 43,7 | 43,7 |
| 2 | 4,86 | 19,5 | 63,2 |
| 3 | 4,08 | 16,4 | 79,5 |
| 4 | 2,22 | 8,9 | 88,5 |
| 5 | 1,59 | 6,4 | 94,8 |

| **III. PCA eigenvectors for Winter** | | | | | |
| --- | --- | --- | --- | --- | --- |
| **Variable** | **PC1** | **PC2** | **PC3** | **PC4** | **PC5** |
| Salinity (psu) | -0,069 | 0,091 | 0,051 | 0,077 | 0,323 |
| NH_4_ (uM) | 0,032 | 0,01 | -0,01 | -0,06 | 0,004 |
| PO_4_ (uM) | 0,188 | -0,39 | -0,371 | 0,249 | 0,438 |
| NO_3_ (uM) | 0,233 | -0,507 | -0,452 | 0,076 | -0,509 |
| NO_2_ (uM) | 0,25 | -0,129 | -0,146 | -0,566 | 0,455 |
| SiO_2_ (uM) | 0,041 | -0,059 | -0,088 | -0,17 | -0,072 |
| Water chl-*a* (ug/l) | -0,5 | -0,147 | -0,279 | -0,085 | 0,078 |
| Water Phaeop. (ug/l) | -0,21 | 0,211 | -0,081 | 0,319 | -0,008 |
| Water CPE (ug/l) | -0,483 | -0,062 | -0,258 | 0,022 | 0,063 |
| Water POC (ug/l) | -0,472 | -0,125 | -0,103 | 0,01 | 0,012 |
| TRIS (uM/gr) | -0,024 | -0,059 | -0,006 | -0,022 | 0,035 |
| Sediment chl-*a* (ug/g) | -0,034 | -0,165 | 0,237 | 0,244 | -0,021 |
| Sediment Phaeop. (ug/g) | -0,076 | -0,324 | 0,281 | 0,069 | 0,199 |
| Sediment CPE (ug/g) | -0,065 | -0,286 | 0,297 | 0,167 | 0,114 |
| Sediment POC (ug/g) | -0,025 | -0,238 | 0,182 | -0,108 | -0,223 |
| % labile OM | -0,066 | -0,231 | 0,235 | -0,047 | -0,143 |
| % refractory OM | -0,047 | -0,194 | 0,195 | -0,093 | 0,093 |
| total OM | -0,061 | -0,226 | 0,229 | -0,068 | -0,051 |
| Temperature (°C) | 0,009 | 0,019 | -0,009 | -0,012 | 0,039 |
| Sediment temperature (°C) | 0,006 | 0,009 | 0 | -0,002 | 0,025 |
| pH | -0,165 | -0,113 | 0,058 | -0,18 | 0,037 |
| O_2_ (mg/lt) | -0,039 | -0,058 | 0,037 | -0,098 | -0,011 |
| Eh (mV) | 0,098 | 0,16 | -0,226 | 0,245 | 0,071 |
| silt & clay (%) | 0,112 | -0,091 | 0,054 | 0,339 | 0,163 |
| sand% | -0,112 | 0,091 | -0,054 | -0,339 | -0,163 |
| water chl-*a*/phaeop. (ug/l) | -0,061 | -0,045 | -0,036 | -0,106 | 0,157 |

| Eigenvalues | |  |  |
| --- | --- | --- | --- |
| PC | Eigenvalues | %Variation | Cum.%Variation |
| 1 | 11,5 | 56,5 | 56,5 |
| 2 | 3,38 | 16,5 | 73 |
| 3 | 1,95 | 9,6 | 82,6 |
| 4 | 1,41 | 6,9 | 89,5 |
| 5 | 0,838 | 4,1 | 93,6 |

| **IV. PCA eigenvectors for Spring** | | | | | |
| --- | --- | --- | --- | --- | --- |
| **Variable** | **PC1** | **PC2** | **PC3** | **PC4** | **PC5** |
| Salinity (psu) | -0,154 | -0,163 | -0,2 | 0,257 | -0,01 |
| NH_4_ (uM) | 0,005 | -0,019 | 0 | -0,017 | -0,042 |
| PO_4_ (uM) | -0,207 | 0,342 | 0,111 | -0,161 | -0,106 |
| NO_3_ (uM) | -0,109 | 0,207 | -0,003 | -0,285 | -0,205 |
| NO_2_ (uM) | 0 | 0,027 | -0,056 | 0,031 | -0,177 |
| SiO_2_ (uM) | 0,196 | 0,082 | 0,195 | -0,328 | -0,174 |
| Water chl-*a* (ug/l) | -0,016 | -0,018 | -0,062 | -0,013 | 0,011 |
| Water Phaeop. (ug/l) | -0,174 | -0,271 | -0,633 | -0,16 | 0,207 |
| Water CPE (ug/l) | -0,065 | -0,094 | -0,237 | -0,058 | 0,07 |
| Water POC (ug/l) | -0,106 | -0,175 | -0,106 | 0,271 | -0,171 |
| TRIS (uM/gr) | 0,043 | -0,462 | 0,227 | 0,32 | -0,523 |
| Sediment chl-*a* (ug/g) | 0,127 | -0,117 | 0,055 | -0,07 | -0,074 |
| Sediment Phaeop. (ug/g) | 0,258 | 0,063 | 0,235 | 0,164 | 0,271 |
| Sediment CPE (ug/g) | 0,226 | -0,02 | 0,175 | 0,067 | 0,133 |
| Sediment POC (ug/g) | 0,228 | -0,158 | 0,104 | -0,289 | 0,223 |
| % labile OM | 0,197 | -0,253 | 0,073 | -0,246 | -0,003 |
| % refractory OM | 0,168 | -0,401 | -0,008 | -0,143 | 0,12 |
| total OM | 0,193 | -0,326 | 0,043 | -0,214 | 0,048 |
| Temperature (°C) | 0,015 | -0,048 | 0,057 | 0,089 | 0,083 |
| Sediment temperature (°C) | -0,005 | 0,004 | 0,015 | 0,073 | 0,085 |
| pH | -0,04 | 0,008 | 0,181 | 0,294 | 0,571 |
| O_2_ (mg/lt) | -0,054 | 0,037 | 0,076 | 0,315 | 0,058 |
| Eh (mV) | -0,075 | 0,069 | -0,112 | -0,108 | 0,063 |
| silt & clay (%) | 0,517 | 0,219 | -0,333 | 0,168 | -0,103 |
| sand% | -0,517 | -0,219 | 0,333 | -0,168 | 0,103 |
| water chl-*a*/phaeop. (ug/l) | 0,01 | 0,007 | 0,001 | 0,002 | 0,006 |

| Eigenvalues | |  |  |
| --- | --- | --- | --- |
| PC | Eigenvalues | %Variation | Cum.%Variation |
| 1 | 6,8 | 46,5 | 46,5 |
| 2 | 2,71 | 18,5 | 65 |
| 3 | 2,04 | 14 | 79 |
| 4 | 1,29 | 8,8 | 87,8 |
| 5 | 0,86 | 5,9 | 93,6 |

| **V. PCA eigenvectors for Summer** | | | | | |
| --- | --- | --- | --- | --- | --- |
| **Variable** | **PC1** | **PC2** | **PC3** | **PC4** | **PC5** |
| Salinity (psu) | -0,12 | -0,298 | 0,404 | 0,02 | -0,052 |
| NH_4_ (uM) | 0,277 | -0,677 | -0,305 | -0,283 | -0,094 |
| PO_4_ (uM) | -0,093 | -0,144 | -0,103 | 0,164 | 0,017 |
| NO_3_ (uM) | -0,08 | 0,074 | -0,228 | 0,208 | -0,054 |
| NO_2_ (uM) | 0,005 | -0,011 | 0,008 | -0,014 | 0,008 |
| SiO_2_ (uM) | 0,092 | 0,117 | -0,638 | -0,16 | -0,136 |
| Water chl-*a* (ug/l) | -0,052 | -0,105 | 0,015 | 0,02 | 0,23 |
| Water Phaeop. (ug/l) | -0,265 | -0,228 | -0,014 | 0,327 | 0,005 |
| Water CPE (ug/l) | -0,121 | -0,155 | 0,008 | 0,113 | 0,196 |
| Water POC (ug/l) | -0,022 | -0,041 | 0,04 | 0,021 | 0,038 |
| TRIS (uM/gr) | -0,049 | -0,09 | -0,246 | 0,538 | 0,28 |
| Sediment chl-*a* (ug/g) | 0,324 | 0,284 | 0,239 | 0,126 | -0,105 |
| Sediment Phaeop. (ug/g) | 0,352 | -0,198 | 0,085 | -0,061 | -0,012 |
| Sediment CPE (ug/g) | 0,385 | 0,02 | 0,174 | 0,026 | -0,06 |
| Sediment POC (ug/g) | 0,315 | 0,035 | -0,119 | 0,187 | 0,146 |
| % labile OM | 0,35 | -0,029 | -0,119 | 0,249 | 0,086 |
| % refractory OM | 0,256 | -0,021 | 0,191 | 0,193 | 0,104 |
| total OM | 0,326 | -0,027 | 0,005 | 0,236 | 0,097 |
| Temperature (°C) | 0,034 | -0,017 | -0,014 | -0,075 | -0,093 |
| Sediment temperature (°C) | 0,024 | 0,013 | -0,022 | -0,01 | -0,007 |
| pH | -0,008 | 0,39 | -0,217 | 0 | 0,12 |
| O_2_ (mg/lt) | 0,139 | 0,184 | 0,003 | -0,153 | -0,221 |
| Eh (mV) | -0,024 | -0,05 | -0,052 | 0,012 | -0,012 |
| silt & clay (%) | 0,037 | 0,043 | 0,021 | -0,295 | 0,572 |
| sand% | -0,037 | -0,043 | -0,021 | 0,295 | -0,572 |
| water chl-*a*/phaeop. (ug/l) | 0,017 | -0,009 | 0,027 | 0,01 | 0,061 |

| Eigenvalues | |  |  |
| --- | --- | --- | --- |
| PC | Eigenvalues | %Variation | Cum.%Variation |
| 1 | 7,85 | 38,5 | 38,5 |
| 2 | 5,36 | 26,3 | 64,8 |
| 3 | 2,42 | 11,9 | 76,7 |
| 4 | 2,02 | 9,9 | 86,6 |
| 5 | 1,52 | 7,5 | 94,1 |

| **VI. PCA eigenvectors for Mazoma** | | | | | |
| --- | --- | --- | --- | --- | --- |
| **Variable** | **PC1** | **PC2** | **PC3** | **PC4** | **PC5** |
| Salinity (psu) | 0,154 | -0,135 | -0,071 | -0,293 | -0,031 |
| NH_4_ (uM) | 0,266 | -0,292 | 0,64 | -0,351 | 0,075 |
| PO_4_ (uM) | 0,11 | 0,39 | 0,057 | -0,126 | 0,211 |
| NO_3_ (uM) | -0,071 | 0,1 | -0,012 | 0,034 | 0,205 |
| NO_2_ (uM) | -0,061 | -0,025 | -0,031 | 0,106 | 0,09 |
| SiO_2_ (uM) | 0,092 | -0,025 | 0,293 | 0,147 | 0,161 |
| Water chl-*a* (ug/l) | -0,451 | -0,227 | 0,101 | -0,205 | -0,102 |
| Water Phaeop. (ug/l) | -0,163 | -0,066 | 0,109 | -0,03 | 0,014 |
| Water CPE (ug/l) | -0,427 | -0,211 | 0,117 | -0,181 | -0,082 |
| Water POC (ug/l) | -0,428 | -0,186 | -0,043 | 0,031 | 0,072 |
| TRIS (uM/gr) | -0,097 | -0,081 | 0,29 | 0,585 | 0,004 |
| Sediment chl-*a* (ug/g) | 0,075 | -0,19 | -0,295 | 0,052 | -0,058 |
| Sediment Phaeop. (ug/g) | 0,161 | -0,393 | -0,113 | -0,217 | 0,161 |
| Sediment CPE (ug/g) | 0,139 | -0,343 | -0,22 | -0,11 | 0,072 |
| Sediment POC (ug/g) | 0,058 | -0,237 | -0,041 | 0,144 | 0,166 |
| % labile OM | 0,08 | -0,254 | 0,049 | 0,255 | 0,231 |
| % refractory OM | 0,103 | -0,214 | -0,114 | 0,238 | 0,018 |
| total OM | 0,093 | -0,248 | -0,017 | 0,26 | 0,152 |
| Temperature (°C) | 0,266 | 0,066 | 0,023 | -0,057 | -0,072 |
| Sediment temperature (°C) | 0,059 | 0,039 | 0,422 | 0,112 | -0,393 |
| pH | -0,227 | 0,029 | -0,045 | -0,035 | -0,173 |
| O_2_ (mg/lt) | -0,127 | 0,026 | 0,003 | 0,11 | 0,008 |
| Eh (mV) | -0,021 | 0,147 | -0,063 | -0,087 | 0,202 |
| silt & clay (%) | 0,151 | -0,108 | -0,108 | 0,069 | -0,49 |
| sand% | -0,151 | 0,108 | 0,108 | -0,069 | 0,49 |
| water chl-*a*/phaeop. (ug/l) | -0,059 | -0,048 | -0,015 | -0,066 | -0,006 |

| Eigenvalues | |  |  |
| --- | --- | --- | --- |
| PC | Eigenvalues | %Variation | Cum.%Variation |
| 1 | 17,4 | 47,3 | 47,3 |
| 2 | 8,45 | 23 | 70,3 |
| 3 | 5,31 | 14,4 | 84,7 |
| 4 | 2,84 | 7,7 | 92,5 |
| 5 | 2 | 5,4 | 97,9 |

| **VII. PCA eigenvectors for Logarou** | | | | | |
| --- | --- | --- | --- | --- | --- |
| **Variable** | **PC1** | **PC2** | **PC3** | **PC4** | **PC5** |
| Salinity (psu) | 0,33 | -0,054 | 0,027 | 0,025 | -0,408 |
| NH_4_ (uM) | 0,005 | -0,017 | 0,008 | 0,066 | -0,072 |
| PO_4_ (uM) | -0,022 | 0,107 | -0,321 | -0,188 | -0,118 |
| NO_3_ (uM) | 0,02 | -0,03 | 0,034 | -0,012 | 0,061 |
| NO_2_ (uM) | -0,139 | 0,004 | 0,083 | -0,121 | -0,123 |
| SiO_2_ (uM) | 0,073 | -0,122 | -0,068 | 0,147 | -0,081 |
| Water chl-*a* (ug/l) | -0,104 | -0,028 | 0,146 | 0,054 | -0,051 |
| Water Phaeop. (ug/l) | -0,397 | -0,1 | 0,172 | 0,127 | -0,351 |
| Water CPE (ug/l) | -0,204 | -0,053 | 0,173 | 0,082 | -0,145 |
| Water POC (ug/l) | -0,273 | 0,039 | 0,012 | -0,107 | 0,223 |
| TRIS (uM/gr) | -0,285 | 0,33 | -0,141 | 0,143 | 0,014 |
| Sediment chl-*a* (ug/g) | 0,135 | 0,206 | 0,37 | 0,353 | 0,29 |
| Sediment Phaeop. (ug/g) | 0,103 | 0,285 | 0,153 | -0,028 | -0,003 |
| Sediment CPE (ug/g) | 0,133 | 0,283 | 0,284 | 0,161 | 0,145 |
| Sediment POC (ug/g) | 0,1 | 0,187 | 0,128 | -0,141 | 0,013 |
| % labile OM | 0,097 | 0,206 | 0,106 | -0,108 | -0,02 |
| % refractory OM | 0,203 | 0,343 | 0,133 | -0,236 | -0,101 |
| total OM | 0,146 | 0,272 | 0,122 | -0,166 | -0,055 |
| Temperature (°C) | 0,304 | 0,064 | -0,396 | 0,143 | -0,242 |
| Sediment temperature (°C) | 0,148 | 0,076 | -0,247 | 0,216 | -0,19 |
| pH | -0,054 | 0,113 | -0,39 | -0,366 | 0,458 |
| O_2_ (mg/lt) | -0,395 | 0,422 | -0,261 | 0,412 | -0,033 |
| Eh (mV) | -0,286 | -0,097 | 0,207 | -0,386 | -0,17 |
| silt & clay (%) | 0,078 | -0,284 | -0,001 | 0,2 | 0,254 |
| sand% | -0,078 | 0,284 | 0,001 | -0,2 | -0,254 |
| water chl-*a*/phaeop. (ug/l) | 0,065 | -0,047 | 0,039 | 0,015 | 0,097 |

| Eigenvalues | |  |  |
| --- | --- | --- | --- |
| PC | Eigenvalues | %Variation | Cum.%Variation |
| 1 | 5,65 | 38,1 | 38,1 |
| 2 | 4,16 | 28 | 66,1 |
| 3 | 3,05 | 20,6 | 86,7 |
| 4 | 0,908 | 6,1 | 92,8 |
| 5 | 0,718 | 4,8 | 97,6 |

| **VIII. PCA eigenvectors for Tsopeli** | | | | | |
| --- | --- | --- | --- | --- | --- |
| **Variable** | **PC1** | **PC2** | **PC3** | **PC4** | **PC5** |
| Salinity (psu) | -0,206 | 0,239 | -0,306 | -0,03 | -0,224 |
| NH_4_ (uM) | -0,083 | -0,024 | -0,183 | -0,085 | -0,059 |
| PO_4_ (uM) | 0,04 | 0,124 | 0,083 | 0,502 | -0,175 |
| NO_3_ (uM) | 0,076 | -0,097 | 0,023 | 0,423 | -0,057 |
| NO_2_ (uM) | 0,362 | -0,524 | -0,09 | 0,015 | -0,179 |
| SiO_2_ (uM) | -0,075 | -0,115 | -0,083 | 0,023 | 0,085 |
| Water chl-*a* (ug/l) | -0,097 | -0,018 | -0,195 | -0,151 | 0,008 |
| Water Phaeop. (ug/l) | -0,51 | 0,012 | 0,452 | 0,143 | -0,156 |
| Water CPE (ug/l) | -0,231 | -0,012 | -0,032 | -0,085 | -0,039 |
| Water POC (ug/l) | 0,01 | -0,008 | 0,108 | -0,07 | 0,011 |
| TRIS (uM/gr) | -0,256 | -0,26 | -0,191 | -0,075 | -0,384 |
| Sediment chl-*a* (ug/g) | 0,058 | -0,076 | 0,089 | -0,076 | -0,149 |
| Sediment Phaeop. (ug/g) | 0,079 | -0,134 | -0,134 | -0,155 | -0,201 |
| Sediment CPE (ug/g) | 0,078 | -0,123 | -0,039 | -0,136 | -0,201 |
| Sediment POC (ug/g) | 0,076 | -0,014 | 0,213 | -0,268 | -0,033 |
| % labile OM | 0,034 | 0,016 | 0,219 | -0,253 | -0,034 |
| % refractory OM | 0,096 | 0,163 | 0,26 | -0,383 | -0,146 |
| total OM | 0,062 | 0,078 | 0,246 | -0,319 | -0,082 |
| Temperature (°C) | -0,174 | 0,344 | 0,03 | -0,01 | 0,096 |
| Sediment temperature (°C) | -0,383 | -0,214 | 0,095 | 0,013 | 0,188 |
| pH | -0,076 | -0,172 | 0,177 | -0,102 | 0,034 |
| O_2_ (mg/lt) | 0,174 | -0,275 | 0,362 | 0,101 | 0,426 |
| Eh (mV) | 0,408 | 0,449 | 0,107 | 0,114 | -0,196 |
| silt & clay (%) | 0,009 | 0,091 | -0,242 | -0,124 | 0,388 |
| sand% | -0,009 | -0,091 | 0,242 | 0,124 | -0,388 |
| water chl-*a*/phaeop. (ug/l) | 0,056 | -0,036 | -0,095 | -0,037 | -0,034 |

| Eigenvalues | |  |  |
| --- | --- | --- | --- |
| PC | Eigenvalues | %Variation | Cum.%Variation |
| 1 | 7,31 | 37,5 | 37,5 |
| 2 | 5,47 | 28,1 | 65,6 |
| 3 | 2,45 | 12,6 | 78,2 |
| 4 | 1,76 | 9 | 87,2 |
| 5 | 1,45 | 7,5 | 94,7 |

| **IX. PCA eigenvectors for Tsoukalio** | | | | | |
| --- | --- | --- | --- | --- | --- |
| **Variable** | **PC1** | **PC2** | **PC3** | **PC4** | **PC5** |
| Salinity (psu) | 0,176 | -0,158 | -0,06 | 0,031 | -0,323 |
| NH_4_ (uM) | -0,033 | 0,006 | -0,027 | -0,031 | -0,029 |
| PO_4_ (uM) | -0,155 | -0,043 | 0,604 | -0,011 | 0,239 |
| NO_3_ (uM) | -0,516 | -0,213 | 0,277 | -0,146 | -0,441 |
| NO_2_ (uM) | -0,356 | -0,039 | -0,062 | -0,185 | -0,039 |
| SiO_2_ (uM) | 0,172 | 0,035 | -0,022 | 0,171 | -0,139 |
| Water chl-*a* (ug/l) | -0,008 | -0,028 | -0,09 | -0,043 | -0,016 |
| Water Phaeop. (ug/l) | -0,066 | 0,076 | -0,099 | -0,019 | 0,02 |
| Water CPE (ug/l) | -0,026 | -0,002 | -0,105 | -0,042 | -0,008 |
| Water POC (ug/l) | -0,047 | -0,064 | -0,124 | -0,048 | 0,02 |
| TRIS (uM/gr) | -0,163 | 0,21 | 0,053 | -0,234 | 0,053 |
| Sediment chl-*a* (ug/g) | 0,217 | 0,033 | 0,195 | -0,121 | -0,601 |
| Sediment Phaeop. (ug/g) | 0,008 | 0,109 | 0,262 | 0,256 | 0,046 |
| Sediment CPE (ug/g) | 0,115 | 0,085 | 0,263 | 0,099 | -0,276 |
| Sediment POC (ug/g) | 0,143 | 0,015 | 0,051 | -0,391 | 0,089 |
| % labile OM | 0,168 | 0,124 | 0,11 | -0,419 | -0,034 |
| % refractory OM | 0,191 | -0,04 | 0,081 | -0,278 | 0,082 |
| total OM | 0,185 | 0,061 | 0,103 | -0,378 | 0,013 |
| Temperature (°C) | 0,295 | 0,117 | 0,095 | 0,22 | 0,038 |
| Sediment temperature (°C) | 0,149 | 0,395 | 0,1 | -0,266 | 0,088 |
| pH | 0,157 | 0,276 | 0,037 | 0,115 | -0,166 |
| O_2_ (mg/lt) | -0,046 | 0,127 | 0,006 | -0,025 | 0,239 |
| Eh (mV) | -0,203 | -0,089 | 0,131 | -0,101 | 0,14 |
| silt & clay (%) | 0,145 | -0,216 | 0,348 | 0,121 | 0,148 |
| sand% | -0,145 | 0,216 | -0,348 | -0,121 | -0,148 |
| water chl-*a*/phaeop. (ug/l) | 0,297 | -0,683 | -0,124 | -0,202 | 0,069 |

| Eigenvalues | |  |  |
| --- | --- | --- | --- |
| PC | Eigenvalues | %Variation | Cum.%Variation |
| 1 | 9,4 | 45,5 | 45,5 |
| 2 | 4,49 | 21,7 | 67,2 |
| 3 | 2,82 | 13,7 | 80,9 |
| 4 | 2,06 | 10 | 90,8 |
| 5 | 1,3 | 6,3 | 97,1 |

| **X. PCA eigenvectors for Rodia** | | | | | |
| --- | --- | --- | --- | --- | --- |
| **Variable** | **PC1** | **PC2** | **PC3** | **PC4** | **PC5** |
| Salinity (psu) | -0,14 | 0,048 | 0,055 | -0,04 | -0,075 |
| NH_4_ (uM) | 0,016 | 0,011 | 0,011 | 0,039 | -0,038 |
| PO_4_ (uM) | -0,13 | -0,021 | 0,038 | -0,158 | 0,12 |
| NO_3_ (uM) | 0,156 | 0,114 | 0,143 | 0,025 | -0,168 |
| NO_2_ (uM) | 0,121 | 0,161 | -0,049 | 0,4 | 0,098 |
| SiO_2_ (uM) | -0,223 | -0,023 | 0,054 | -0,415 | 0,001 |
| Water chl-*a* (ug/l) | -0,004 | -0,009 | 0,034 | 0,035 | -0,124 |
| Water Phaeop. (ug/l) | 0,042 | 0,003 | 0,027 | 0,038 | -0,115 |
| Water CPE (ug/l) | 0,009 | -0,007 | 0,036 | 0,041 | -0,138 |
| Water POC (ug/l) | 0,002 | 0,037 | -0,033 | 0,101 | 0,053 |
| TRIS (uM/gr) | 0,162 | -0,136 | 0,166 | 0,022 | -0,659 |
| Sediment chl-*a* (ug/g) | -0,144 | -0,185 | 0,372 | 0,33 | -0,043 |
| Sediment Phaeop. (ug/g) | -0,018 | -0,333 | -0,001 | 0,04 | -0,25 |
| Sediment CPE (ug/g) | -0,084 | -0,302 | 0,188 | 0,193 | -0,178 |
| Sediment POC (ug/g) | -0,332 | 0,017 | -0,148 | 0,093 | -0,221 |
| % labile OM | -0,355 | -0,047 | -0,039 | 0,177 | -0,174 |
| % refractory OM | -0,377 | -0,131 | 0,177 | 0,189 | 0,319 |
| total OM | -0,38 | -0,084 | 0,049 | 0,19 | 0,025 |
| Temperature (°C) | -0,184 | -0,153 | 0,031 | -0,435 | 0,011 |
| Sediment temperature (°C) | -0,09 | -0,111 | 0,065 | -0,302 | -0,072 |
| pH | 0,187 | -0,335 | 0,56 | -0,033 | 0,368 |
| O_2_ (mg/lt) | 0,307 | -0,101 | 0,043 | 0,022 | 0,063 |
| Eh (mV) | 0,088 | 0,157 | -0,112 | 0,268 | 0,117 |
| silt & clay (%) | 0,076 | -0,483 | -0,424 | 0,061 | 0,095 |
| sand% | -0,076 | 0,483 | 0,424 | -0,061 | -0,095 |
| water chl-*a*/phaeop. (ug/l) | -0,325 | 0,161 | -0,121 | 0,051 | 0,05 |

| Eigenvalues | |  |  |
| --- | --- | --- | --- |
| PC | Eigenvalues | %Variation | Cum.%Variation |
| 1 | 13,6 | 47,7 | 47,7 |
| 2 | 6,71 | 23,6 | 71,3 |
| 3 | 3,49 | 12,3 | 83,6 |
| 4 | 2,82 | 9,9 | 93,5 |
| 5 | 0,841 | 3 | 96,4 |
